# Aberrant DNA methylation of *Tgfb1* in diabetic kidney mesangial cells

**DOI:** 10.1038/s41598-018-34612-3

**Published:** 2018-11-05

**Authors:** Shigeyoshi Oba, Nobuhiro Ayuzawa, Mitsuhiro Nishimoto, Wakako Kawarazaki, Kohei Ueda, Daigoro Hirohama, Fumiko Kawakami-Mori, Tatsuo Shimosawa, Takeshi Marumo, Toshiro Fujita

**Affiliations:** 1 Division of Clinical Epigenetics, Research Center for Advanced Science and Technology, The University of Tokyo, Tokyo, Japan; 2 Division of Endocrinology, Mitsui Memorial Hospital, Tokyo, Japan; 3 Department of Clinical Laboratory, International University of Health and Welfare, School of Medicine, Mita Hospital IUHW, Tokyo, Japan

## Abstract

Epigenetic modulation may underlie the progression of diabetic nephropathy (DN). Involvement of TGFB1 in mesangial fibrosis of DN led us to hypothesize that *Tgfb1* DNA demethylation contributes to progression of DN. In primary mesangial cells from diabetic (*db/db*) mouse kidneys, demethylation of *Tgfb1* DNA and upregulation of *Tgfb1* mRNA progressed simultaneously. USF1 binding site in *Tgfb1* promoter region were demethylated, and binding of USF1 increased, with decreased binding of DNMT1 in *db/db* compared with control. Given downregulation of *Tgfb1* expression by folic acid, antioxidant Tempol reversed DNA demethylation, with increased and decreased recruitment of DNMT1 and USF1 to the promoter, resulting in decreased *Tgfb1* expression in *db/db* mice. Addition of H_2_O_2_ to mesangial cells induced DNA demethylation and upregulated *Tgfb1* expression. Finally, Tempol attenuated mesangial fibrosis in *db/db* mice. We conclude that aberrant DNA methylation of *Tgfb1* due to ROS overproduction play a key to mesangial fibrosis during DN progression.

## Introduction

Diabetic nephropathy (DN) is the most frequent cause of end-stage renal disease (ESRD) in developed countries^1^, and population of patients with DN continues to increase, despite advanced management of diabetes^2^, possibly because of the irreversibility of DN^3,4^. Epigenetic mechanisms have key roles in the persistent phenotypic changes of blood vessels and organs^5,6^ that result in diabetes-related complications, including DN^7^. Indeed, DNA methylation changes have been observed in the kidneys of a mouse model of renal fibrosis^8^; however, the DNA methylation status of whole kidney represents the sum of the methylation of all of the dozens of different cell types that compose the organ. DNA methylation is cell type-specific and differs among individual cells^9^. We recently evaluated DNA methylation in proximal tubular cells purified from the kidneys of diabetic mice, and found aberrant DNA methylation, leading to persistent mRNA expression of DN-related genes^10^.

The prominent role of TGFB1 in renal fibrosis has been demonstrated since the first report indicating the amelioration of experimental renal fibrosis by neutralization of this protein^11^. Subsequently, several investigators have demonstrated that levels of TGFB1 and its downstream signaling pathways are enhanced in renal cells during DN progression^12,13^ and that TGFB1 has a critical role in mesangial fibrosis, a histological change typical of DN, under diabetic conditions by inducing the expression of extracellular matrix proteins, including collagen^14–16^.

DNA demethylation at promoter regions leads to enhanced gene expression; DNA methylation is recognized by methyl-binding proteins that can recruit transcription factors, and other proteins via protein-protein interactions, to alter mRNA expression levels^17^. Several transcription factors are involved in transcription of *Tgfb1* induced by glucose and angiotensin II^18,19^. Given the presence of specific binding sites for USF1^20,21^ and SREBP1^22,23^ within the *Tgfb1* promoter, we aimed to clarify the relationship between the binding of USF1 and SREBP1 to their corresponding binding sites and DNA methylation status at CpG sites in *Tgfb1* promoter in diabetic mice.

During irreversible DN, diabetes induces aberrant DNA methylation and concomitant changes in mRNA expression, including of key genes implicated in glucose metabolism and transport, in proximal tubules of diabetic kidneys, leading to resistance to the effects of antidiabetic drugs^10^. However, elevated glucose levels are not the sole factor contributing to maladaptive epigenetic modifications in diabetes. Hyper-methylation of the *PPARGC1* gene promoter observed in skeletal muscle of obese patients can be reversed to the levels seen in non-obese individuals after weight reduction by bariatric surgery, possibly through the reversal of obesity-induced metabolic abnormalities^24^. DNA methylation can also be influenced by reactive oxygen species (ROS), both directly through oxidative modification preventing DNA methylation, and indirectly through its effects on methylation writing/erasing enzymes^25,26^. Overproduction of ROS in the kidney can induce diabetic glomerular injury and mesangial fibrosis through TGFB1 upregulation in DN^27–29^; however, treatment with Tempol, a superoxide dismutase (SOD) mimetic, can decrease *Tgfb1* mRNA, inhibit mesangial cell fibrosis, and decrease albuminuria in diabetic rodents^30,31^.

In the present study, to evaluate the mesangial cell-specific DNA methylation status, we purified mesangial cells by culturing glomerular cells sieved from the kidneys of diabetic (*db/db*), and control non-diabetic (*db/m*), mice. This is the first study to demonstrate aberrant DNA methylation in mesangial cells from diabetic mice. To evaluate the reversibility of the DNA methylation changes observed in DN, and the sequence of events leading to diabetic kidney, here we studied the effects of anti-oxidant therapy on DNA methylation, *Tgfb1* mRNA expression, and mesangial fibrosis in diabetic *db/db* mice. Our results clearly demonstrate that DNA demethylation of the *Tgfb1* gene, induced by overproduction of ROS, is important for upregulation of *Tgfb1* mRNA and consequent mesangial fibrosis and matrix expansion during DN progression.

## Results

### Analysis of DNA methylation in mesangial cells from a diabetic mouse model

**Figure 1:**
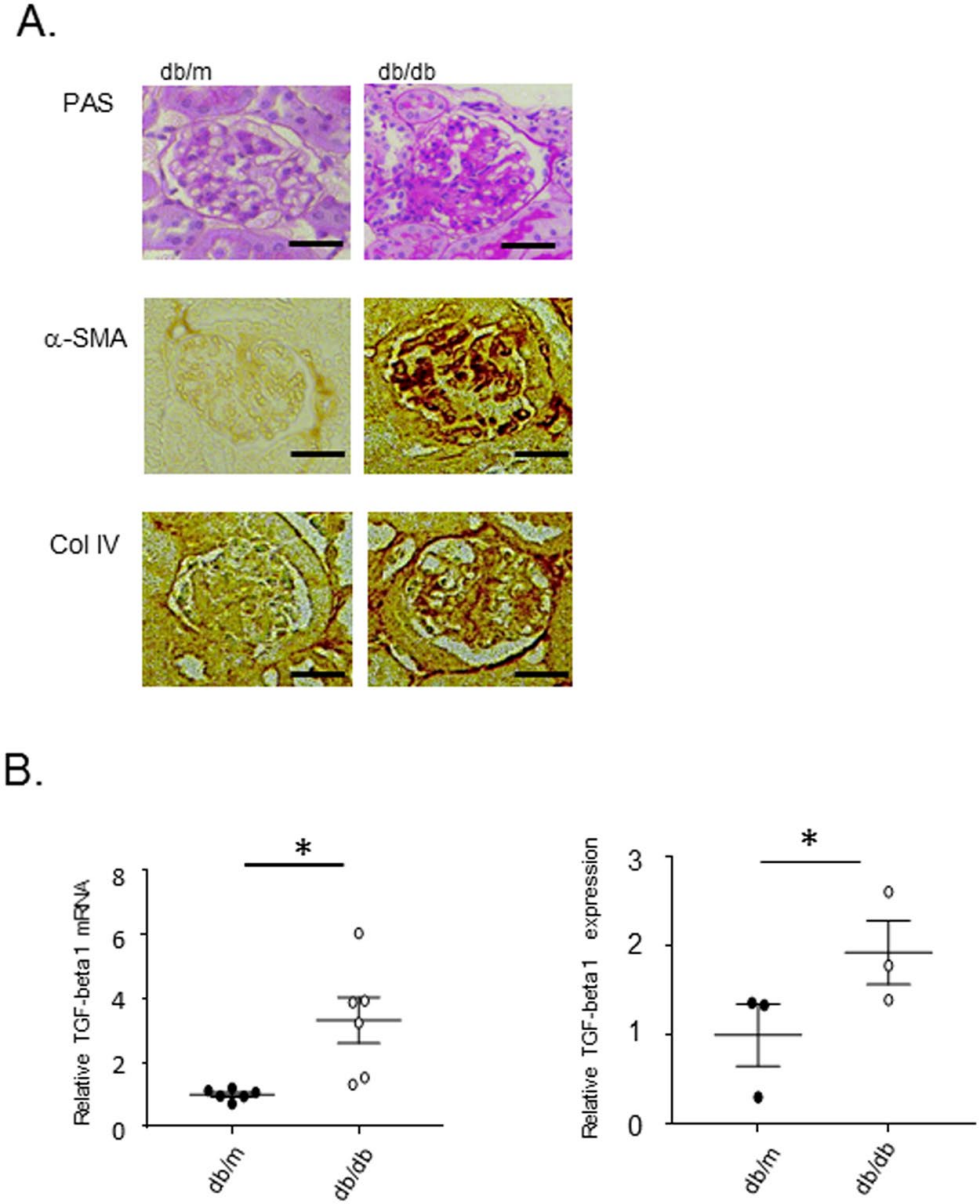
Mesangial cell fibrosis and upregulation of TGFB1 expression in *db/db* mice. (**A**) PAS staining and representative images of immunostaining of α−smooth muscle actin (α-SMA) and type IV collagen (Col IV) in glomeruli from 15-week-old *db/m*, *db/db*, Mesangial cell fibrosis and the expression of α-SMA and Col IV are significantly increased in *db/db* mice compared to *db/m* mice. (**B**) Real-time PCR of *Tgfb1* mRNA (normalized to *Gapdh*) and the results of immunoblotting of TGFB1 (normalized to beta-actin) in whole kidneys of 15-week-old *db/m*, *db/db* mice. The expression of *Tgfb1* mRNA and TGFB1 protein in *db/db* mice significantly higher than that in *db/m* mice. Data represent the mean ± SEM. n = 6. Filled circles: *db/m* mice; open circles: *db/db* mice. Original gel image of western blot analysis are presented in Supplemental Fig. 4.

**Figure 2:**
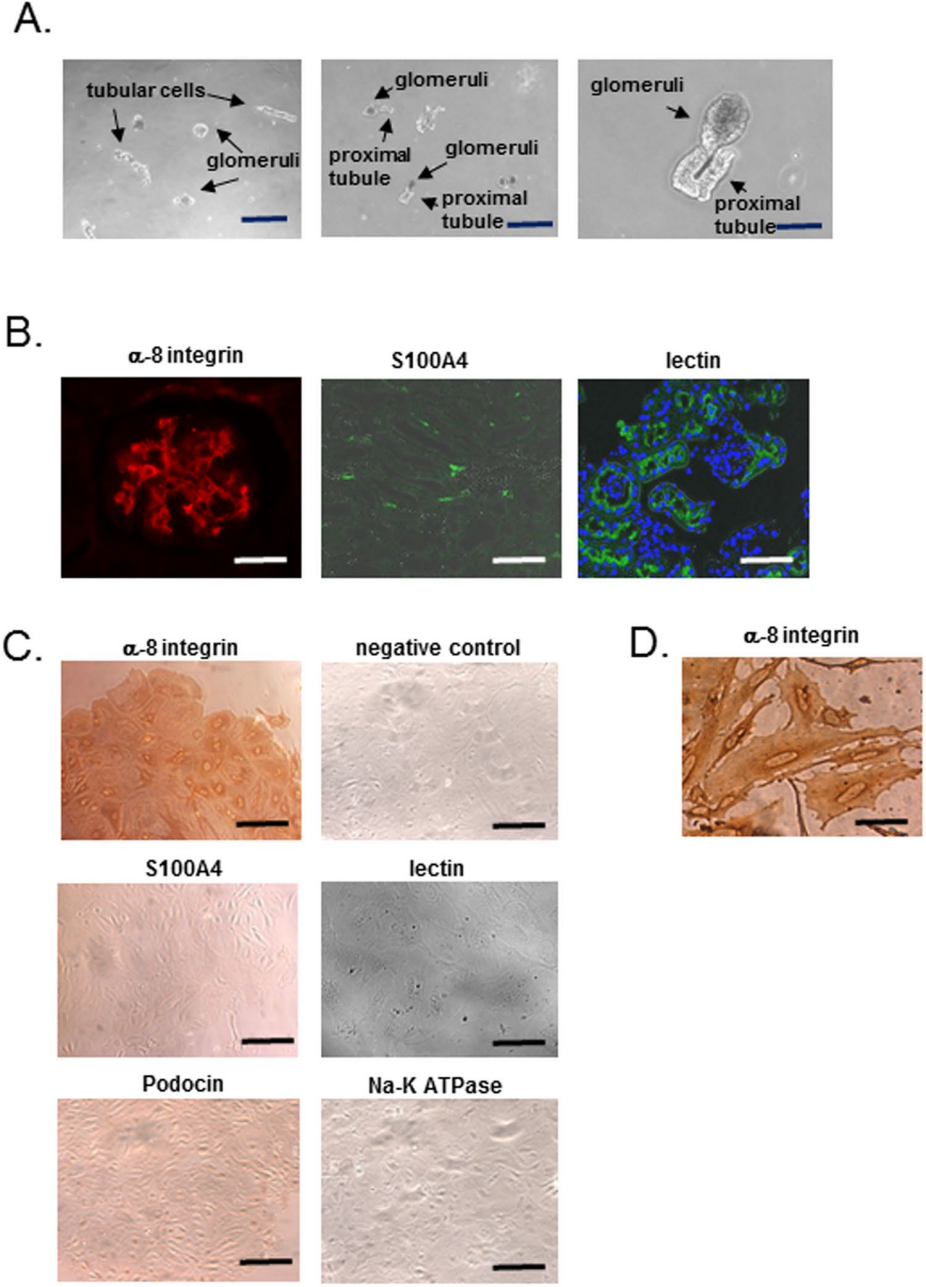
Characterization of primary culture cells. (**A**) The observation of sieving sample under phase-contrast microscope. The sieving sample contains glomeruli and other cells such as tubular cells. Right panel shows sieving sample containing glomeruli connected to proximal tubules. Scale bar, 20 µm (left and middle panel), 8 µm (right panel). (**B**) Immunofluorescent staining of α-8 integrin (mesangial cell marker) and S100A4 (fibroblast marker) in kidneys of C57BL/6 J mice. A glomerulus contains cells with positive staining for α-8 integrin, and some cells with positive staining for S100A are observed in glomeruli and tubulo-interstitial cells. Right panel shows proximal tubular cells and their cells invaginated into parietal layer of Bowman’s capsule with the positive staining for lotus tetragonolobus lectin. Scale bar, 20 µm (left panel), 7 µm (middle and right panel). (**C**) Immunohistochemistry of α-8 integrin, S100A4 with the negative control, podocin (podocyte marker), Na-K ATPase (tubular cell marker) and fluorescence of lotus tetragonolobus lectin (proximal tubular cell marker) in mesangial primary culture cells. All most of the primary cultured cells exhibit positive staining for α-8 integrin but negative staining for the other markers. Scale bar, 20 µm. (**D**) High magnification of α-8 integrin staining. The cells after primary culture included an irregular shape and flattened-cylinder-like cell bodies, typical of mesangial cells. Scale bar, 4 µm.

PAS staining and immunohistochemistry revealed that fibrotic change occurred in mesangial cells of 15-week-old diabetic (*db/db*) mice (Fig. 1A), with the significant increase in expressions of *Tgfb1* mRNA and TGFB1 in the kidney (Fig. 1B). To evaluate the involvement of DNA methylation in the expression of fibrosis related genes mRNAs in mesangial cells, we first characterized mesangial cells purified by primary culture of glomerular cells sieved from the kidneys of a diabetes model mouse. Glomerular cells immediately after sieving include, not only mesangial cells, but also other glomerular cells and proximal tubules, which can invaginate into the mouse glomerulus (Fig. 2A); however, after primary culture, proximal tubular cells are not present; neither primary culture cells show staining for lotus tetragonolobus lectin which is a marker for proximal tubular cells (Fig. 2B,C). Consistent with previous reports using the same sieving method^32^, our primary culture cells exhibited positive staining for integrin subunit α 8 (α-8 integrin/ITGA8, a mesangial cell marker)^33^ and negative staining for podocin (a podocyte marker), S100A4 (a fibroblast marker) and Na-K-ATPase (a tubular cell maker) (Fig. 2C). In addition to the ITGA8-positive stain, the histological characteristics of the cells after primary culture included an irregular shape and flattened-cylinder-like cell bodies, typical of mesangial cells (Fig. 2D). Based on this conclusive evidence that almost all of the primary cultured cells derived using this method are mesangial cells, genomic DNA and mRNA were extracted from primary cultures of mesangial cells from 15-week-old diabetic (*db/db*) mice for further experiments.

**Figure 3:**
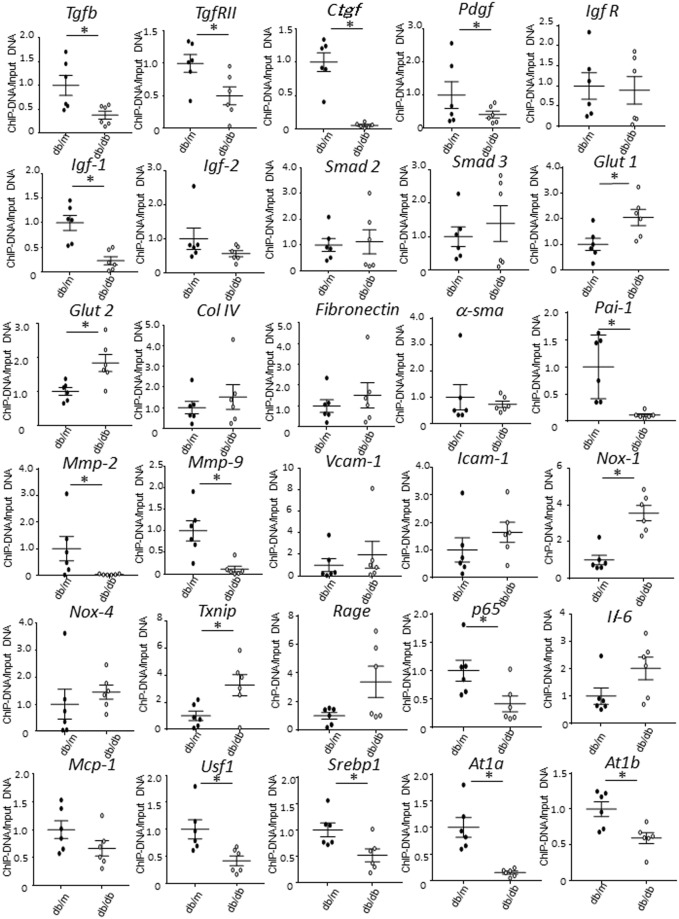
Change of DNA methylation status of fibrosis related genes in mesangial cells of *db/m* and *db/db* mice. Data represent the mean ± SEM. n = 6. Filled circles: *db/m* mice; open circles: *db/db* mice.

At first, we analyzed DNA methylation of fibrosis-related genes using a MethylCollector Ultra kit (Fig. 3). Among these genes, growth factor related genes such as*Tgfb1*, *TgfrII*, *Ctgf*, *Pdgf* and *Igf-1* were predominantly demethylated (Fig. 3 and Table 1). Based upon the previous reports indicating that TGFB1 had a critical role in mesangial fibrosis, we further investigated the DNA demethylation of *Tgfb1* gene in primary culture mesangial cells.

**Table 1:** A list of the change of DNA methylation status of fibrosis related genes of db/db mice.

Gene	DNA Methylation
**Growth Factor**	
*Tgfb1*	↓
*TgfrII*	↓
*Ctgf*	↓
*Ppdgf*	↓
*Igf-1*	↓
*Igf-2*	n.s.
*Igf biding protein 1*	n.s.
*Igf R*	n.s.
*Smad 2*	n.s.
*Smad 3*	n.s.
*Glut 1*	↑
*Glut 2*	↑
*Col IV*	n.s.
*Fibronectin*	n.s.
*α-sma*	n.s.
*Pai-1*	↓
*Mmp-2*	↓
*Mmp-9*	↓
*Vcam-1*	n.s.
*Icam-1*	n.s.
**Reactive oxygen species**	
*Nox-1*	↑
*Nox-4*	n.s.
*Txnip*	↑
*Rage*	n.s.
**Inflammation**	
*p65*	↓
*Il-6*	n.s.
*Mcp-1*	n.s.
*Usf1*	↓
*Srebp1*	↓
*At1a*	↓
*At1b*	↓

**Figure 4:**
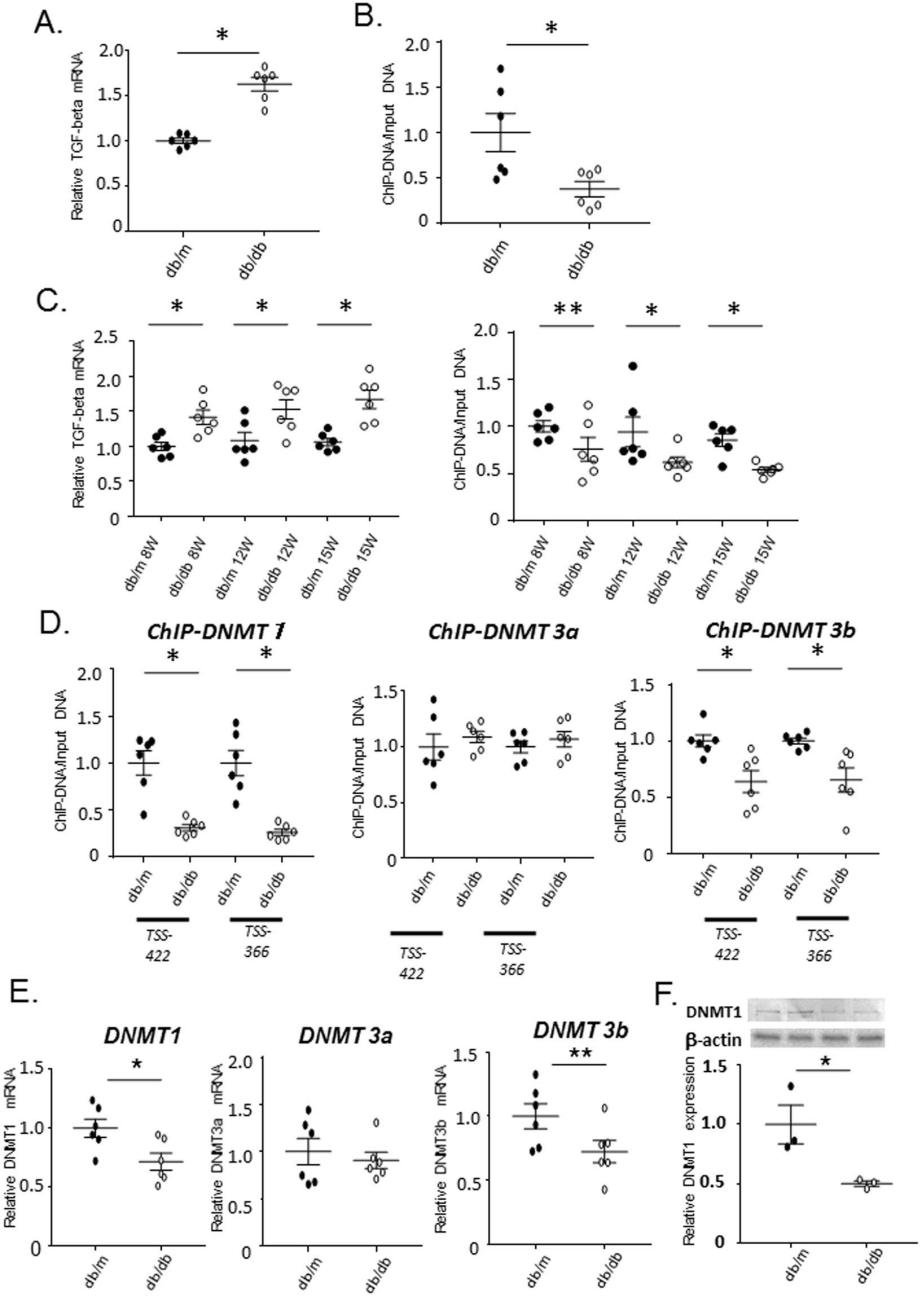
DNA demethylation and upregulation of *Tgfb1* in mesangial cells of *db/db* mice. (**A**) Real-time PCR of *Tgfb1* mRNA (normalized to *Gapdh*) in primary cultured mesangial cells from *db/m* and *db/db* mice. Open circle: The expression of *Tgfb1* mRNA in *db/db* mice significantly higher than that in *db/m* mice. Data represent the mean ± SEM. n = 6. Filled circles: *db/m* mice; open circles: *db/db* mice. (**B**) Quantitation of DNA methylation (using a MethylCollector Ultra kit) in mesangial cells from 15-week-old *db/m* controls and *db/db* mice. Results of amplification from the −422 bp relative to the transcription start site (TSS) regions are shown. DNA methylation of *db/db mice* is significantly lower than that of *db/m mice*. n = 6. (**C**) Time course real-time PCR experiments demonstrating *Tgfb1* mRNA (normalized to *Gapdh*) expression and quantitation of DNA methylation using a MethylCollector Ultra kit in 8-, 12-, and 15-week-old (8 W, 12 W, and 15 W, respectively) *db/m* and *db/db* mice. Following the significant difference in *Tgfb1* mRNA at 8 week and the slight, but insignificant, decrease in DNA methylation in mesangial cells of *db/db* mice at 8 weeks, both parameters at 12 and 15 weeks significantly differed between *db/m* and *db/db* mice. n = 6. (**D**) ChIP analysis of DNMTs in mesangial cells of *db*/*db* mice. The results of ChIP assays for DNMT1, DNMT3A, and DNMT3B in mesangial cells from 12-week-old *db/m* and *db/db* mice at the positions 422 and 366 bp upstream of the TSS. The binding of DNMT1 and DNMT3B to the *Tgfb1* promoter region is significantly decreased in *db/db mice* as compared to *db/m* mice, despite no difference of DNMT3A between both mouse strains. n = 6. (**E**) Real-time PCR of DNMTs mRNA (normalized to *Gapdh*) of DNMT1 in primary cultured mesangial cells from *db/m* and *db/db* mice. The expression of DNMT1 mRNA significantly and DNMT3b mRNA tends to decreased in *db/db mice* as compared to *db/m* mice. Data represent the mean ± SEM. real-time PCR; n = 6, Filled circles: *db/m* mice; open circles: *db/db* mice. (**F**) Western blot analysis of DNMT1 in primary cultured mesangial cells from *db/m* and *db/db* mice. Open circle: The expression of DNMT1 protein significantly decreased in *db/db mice* as compared to *db/m* mice. Data represent the mean ± SEM. n = 3. Filled circles: *db/m* mice; open circles: *db/db* mice. Original gel image of western blot analysis is presented in Supplemental Fig. 3.

Quantitative RT-PCR analysis indicated that the expression of *Tgfb1* mRNA in mesangial cells from *db/db* mice was significantly elevated compared with that in control *db/m* mice (Fig. 4A). DNA methylation analysis of the *Tgfb1* promoter demonstrated that the promoter region was demethylated at positions −422, −366, +121 and +181 bp of the transcription start site (TSS) in mesangial cells from *db/db* mice relative to those from *db/m* mice (Fig. 4B and Supplemental Fig. 1). DNA methylation of the promoter in cells after sieving alone, which comprise mesangial cells and other glomerular cells, were comparable between *db/db* and *db/m* mice (Supplemental Fig. 2)

To evaluate the relationship between the expression of *Tgfb1* mRNA and DNA methylation, we investigated the time course of these parameters (Fig. 4C). Both the expression of *Tgfb1* mRNA and the status of DNA methylation at 8, 12 and 15week significantly differed between db/db and db/m mice, and these parameters tended to simultaneously increase and decrease in a time-dependent manner, respectively.

### ChIP analysis of DNA methyltransferase enzymes in *db/db* mesangial cells

As methyltransferase enzymes are modulators of DNA methylation, ChIP assays to evaluate the roles of DNMT1, DNMT3A, and DNMT3B were performed to clarify the mechanism of DNA demethylation of the *Tgfb1* promoter region in mesangial cells of *db/db* mice. Binding of DNMT1 and DNMT3B to the *Tgfb1* promoter region was substantially decreased in *db/db*, relative to control mice in these cells, while that of DNMT3A was not (Fig. 4D). The observation that the decrease in DNMT1 binding to the promoter was greater than that of DNMT3B led us to hypothesize that the release of DNMT1 from the *Tgfb1* promoter may be the main factor in inducing the observed DNA demethylation in *db/db* mice. Of note, DNMT1 is known to function in the maintenance of DNA methylation^17^.

Noteworthy, the expression of DNMT1 mRNA was significantly decreased and DNMT3b tended to be reduced in diabetic mesangial cells (Fig. 4E). Consistent with decreased DNMT1 mRNA, DNMT1 protein significantly decreased in diabetic mesangial cells (Fig. 4F and Supplemental Fig. 3), in contrast to the increased expression of DNMT1 in the whole kidney of *db/db* mice (Supplemental Fig. 4), as previously reported^34^ and comparable expression in just sieving sample of *db/db* mice (Supplemental Fig. 5).

### Investigation of DNA methylation by bisulfite sequence analysis

**Figure 5:**
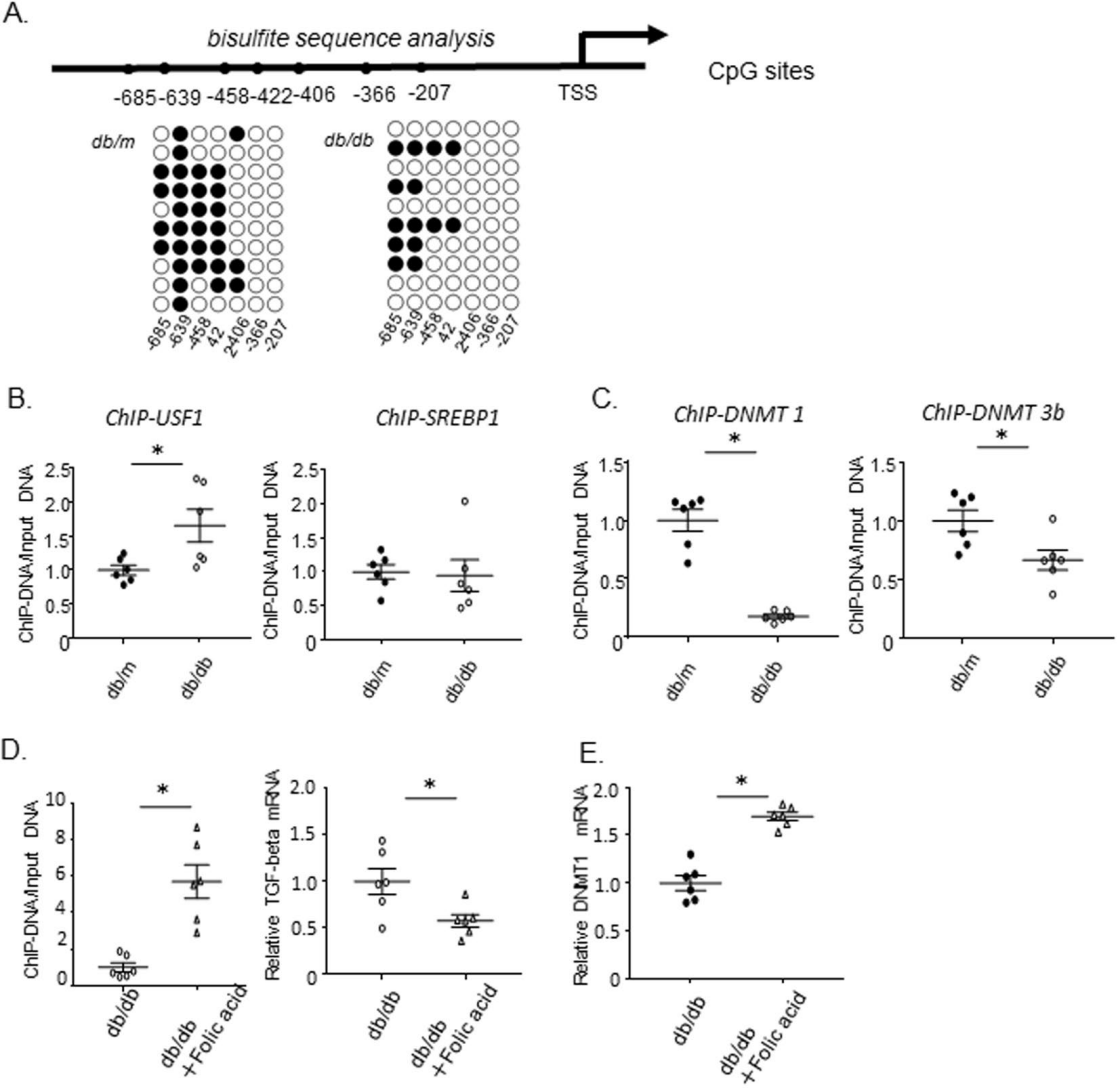
Bisulfite sequence analysis of the *Tgfb1* promoter region, and ChIP assays of USF1 and SREBP1. (**A**) Bisulfite sequence analysis of the *Tgfb1* promoter region in mesangial cells from 15-week-old *db/m* and *db/db* mice. Top, schema of the *Tgfb1* promoter. The dashes and numbers indicate the positions of the cytosine residues of CpG dinucleotides relative to the transcription start site (TSS, +1). Bottom, DNA methylation status of the CpG sites between −207 and −685 bp relative to the TSS. CpGs at positions 639, 458, 422, and 406 bp upstream of the TSS are demethylated in *db/db* mice, while CpGs at positions −366 and −207 were comparably demethylated in both mouse strains. Filled circles represent methylated and open circles demethylated CpG sites. (**B**) The results of ChIP assays of USF1 binding to the site, TSS −641 to −636, and SREBP1 binding to the site, TSS −200 to −32, in mesangial cells from 12-week-old *db/m* and *db/db* mice. The binding of USF1 to its recognition site *in Tgfb1* promoter region (−641 to −636 bp) is significantly increased in *db/db* mice compared with *db/m* mice, but the binding of SREBP1 to its recognition site (−232 to −220 bp) is comparable between *db/m* and *db/db* mice. Data represent the mean ± SEM. n = 6. Filled circles: *db/m* mice; open circles: *db/db* mice. (**C**) The results of ChIP assays of DNMT1 and DNMT3B binding to the USF1 recognition site (TSS −641 to −636) in mesangial cells from 12-week-old *db/m* and *db/db* mice. The binding of DNMT1 and DNMT3B to the corresponding promoter region are significantly decreased in *db/db* mice; the greater binding of DNMT1 than that of DNMT3B. Data represent the mean ± SEM. n = 6. (**D**) Effect of folic acid on DNA methylation and *Tgfb1* mRNA in mesangial cells. Quantitation of DNA methylation in mesangial cells from 15-week-old *db/db* and *db/db* mice treated with Folic acid for 8 weeks revealed tha DNA methylation of *db/db mice* with Folic acid is significantly higher than that of *db/m mice*. Results of amplification from the −639 bp relative to the transcription start site (TSS) regions are shown. Real-time PCR of *Tgfb1* mRNA (normalized to *Gapdh*) in primary cultured mesangial cells from *db/db* and *db/db* mice treated with Folic acid. The expression of *Tgfb1* mRNA in *db/db* mice with Folic acid was significantly lower than that in *db/db* mice. Data represent the mean ± SEM. n = 6. Open circles: *db/db* mice; open triangles: *db/db* mice treated with Folic acid. (**G**) Effect of folic acid on DNMT1 mRNA in mesangial cells. Real-time PCR of DNMT1 mRNA (normalized to *Gapdh*) in primary cultured mesangial cells from *db/db* and *db/db* mice treated with Folic acid. The expression of *Tgfb1* mRNA in *db/db* mice with Folic acid was significantly higher than that in *db/db* mice. Data represent the mean ± SEM. n = 6. Open circles: *db/db* mice; open triangles: *db/db* mice treated with Folic acid.

To more precisely evaluate the position of DNA demethylation in the *Tgfb1* promoter in *db/db* mesangial cells, we performed bisulfite sequence analysis. Figure 5A shows DNA methylation of CpG islands upstream of the *Tgfb1* gene; CpGs at positions 639, 458, 422, and 406 bp upstream of the TSS appeared to be demethylated in *db/db* mice compared with *db/m* mice, whereas CpGs at positions −366 and −207 were comparably demethylated in both mouse strains (Fig. 5A). There are specific binding sites for USF1 and SREBP1 in the *Tgfb1* promoter at positions 641–636 and 220–32 bp upstream of the TSS, respectively^20–22^. The results of our bisulfite sequence analysis indicated that the CpG at position −639, within the USF1-binding site, was fully methylated in DNA from mesangial cells from control *db/m* mice, but half demethylated in *db/db* mice. By contrast, the CpGs at sites (−366 and −207 bp) flanking the SREBP1-binding site (−220 to −32 bp) were demethylated in both control and *db/db* mice (Fig. 5A).

### ChIP analysis of USF1 and SREBF1 binding to the *Tgfb1* promoter

Consistent with the DNA methylation status in each binding site, ChIP analysis revealed that the binding of USF1 to its recognition site in the *Tgfb1* promoter region (−641 to −636 bp) was increased in mesangial cells from *db/db* mice compared with those from *db/m* controls (Fig. 5B). By contrast, the binding of SREBP1 to its recognition site (−220 to −32 bp) was comparable between *db/m* and *db/db* mice. This finding strongly suggests a close relationship between DNA demethylation at the specific USF1-binding site and USF1 occupation of this site during *Tgfb1* transcription. Moreover, supporting this interpretation, the binding of DNMT1 and DNMT3B to the corresponding promoter region (−641 to −636) was significantly decreased (Fig. 5C) and the decrease in the binding of DNMT1 was greater than that of DNMT3B (Fig. 5C). Notably, the decrease in the binding of DNMT1 was greater than that of DNMT3B, consistent with the results of the ChIP assays of DNMT1 and DNMT3B binding to the CpGs, along with observed DNA demethylation (Fig. 4D). Together, these results led us to hypothesize that DNA demethylation due to release of DNMT1 from the promoter, along with recruitment of USF1 to the promoter, contribute to the enhanced transcription of the *Tgfb1* gene in mesangial cells of *db/db* mice.

In order to further investigate the correlation between the binding activities of USF1 and SREBP1 and their expression levels, we measured mRNA and protein of these transcription factors. Both the expression of *Usf1*mRNA and *Srebp1* mRNA in mesangial cells, and protein of USF1 and SREBP1 in kidneys were significantly increased in *db/db* mice compared to *db/m* mice (Supplemental Fig. 6). Indeed, USF1 expressions correlated to its binding acidity, but SREBP1 did not. The causal relationship between expressions of transcriptional factors and their binding activities on the promoter of certain genes remain unknown.

### Folic acid could prevent DNA demethylation of *Tgfb1* and upregulation of TGFB1 expression

To further confirm that the upregulation of TGFB1 expression was induced by DNA demethylation of *Tgfb1* promoter, we studied the effect of treatment with folic acid, which could induce global DNA methylation^35–37^, in the drinking water on *Tgfb1* mRNA expression in *db/db* mice. Folic acid treatment could prevent DNA demethylation of *Tgfb1* and result in downregulation of TGFB1 expression in mesangial cells of *db/db* mice (Fig. 5D), associated with upregulation of DNMT1 mRNA expression in mesangial cells (Fig. 5E).

### Upregulation of ROS in *db/db* mice and the effect of Tempol

**Figure 6:**
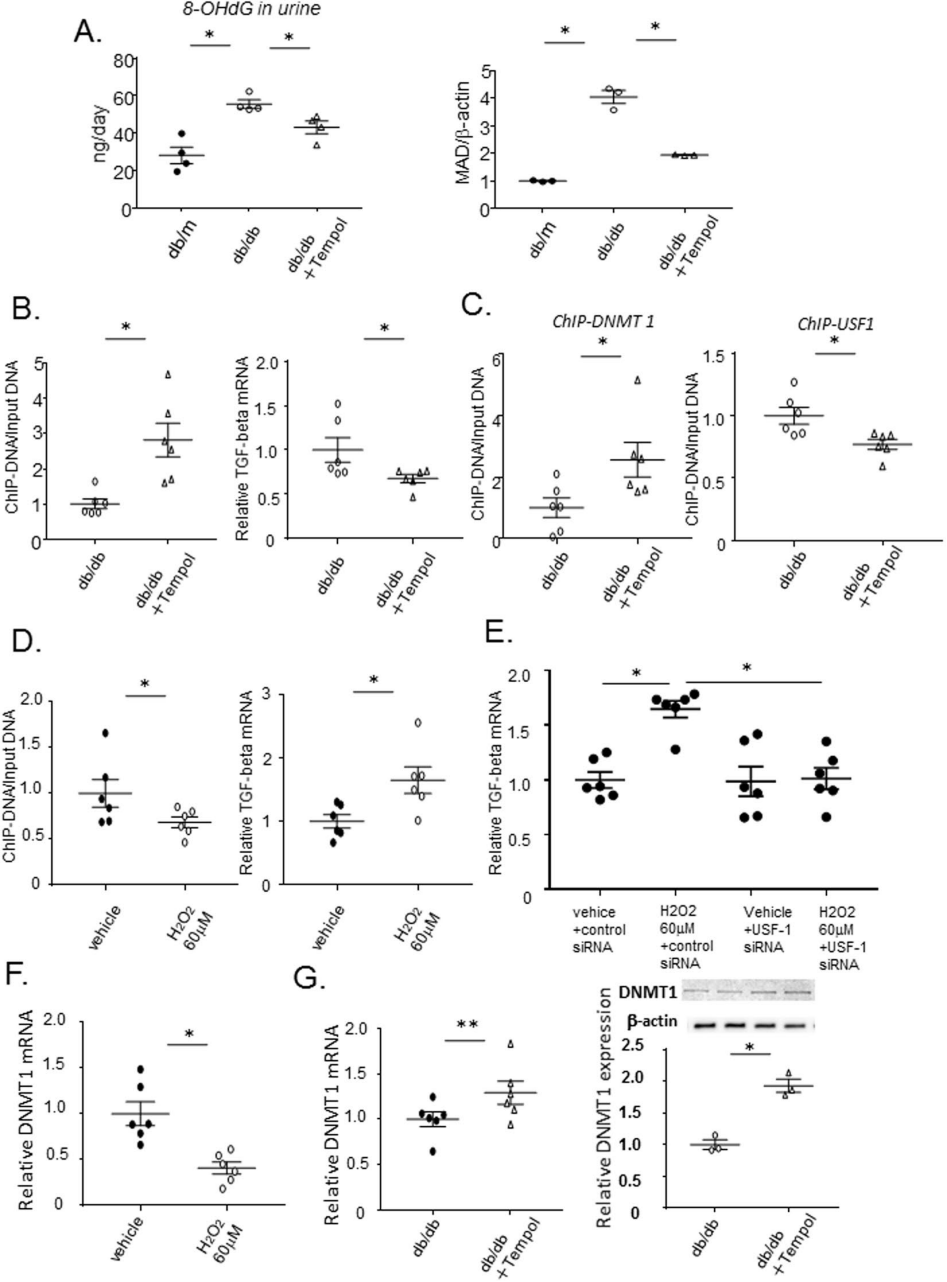
The effect of treatment with the anti-oxidant, Tempol to demethylauin of *Tgfb1* in db/db mice and reactive oxygen species to methylation of *Tgfb1* in mesangial cells. (**A**) Twenty-four hour urine samples were collected from *db/m*, *db/db*, and *db/db* mice treated with Tempol for 8 weeks in metabolic cages, and 8-OHdG was measured using a New 8-OHdG Check ELISA kit. Immunoblotting of MAD in whole kidneys of *db/m*, *db/db*, and *db/db* mice treated with Tempol for 8 weeks. Urinary 8-OHdG and renal MAD content were significantly increased in *db/db* mice compared with *db/m* mice, but treatment with Tempol significantly decreased these parameters. Beta-actin was used for normalization. Data represent the mean ± SEM. 8-OHdG, n = 4; MAD, n = 3. Filled circle: *db/m* mice; open circle: *db/db* mice; open triangle: *db/db*+ Tempol. (**B**) DNA methylation and down-regulation of *Tgfb1* in mesangial cells from *db/db* mice treated with Tempol. Quantitation of DNA methylation, using a MethylCollector Ultra kit, in mesangial cells from 15-week-old *db/db* mice, treated or not treated with Tempol for 8 weeks. Quantitative real-time PCR of *Tgfb1* and *Gapdh* mRNA from 15-week-old *db/db* mice treated or not treated with Tempol for 8 weeks. Tempol significantly increase DNA methylation and decreased *Tgfb1* mRNA. Data represent the mean ± SEM. n = 6. (**C**) The results of ChIP assays to determine binding of DNMT1 and USF1 to the *Tgfb1* promoter (TSS −641 to −636) in mesangial cells from 15-week-old *db/db* mice treated or not treated with Tempol for 8 weeks. Tempol significantly increase and decrease the binding of DNMT1 and USF1 to their respective binding sites. Data represent the mean ± SEM. n = 6. (**D**) Quantitation of DNA methylation, using a MethylCollector Ultra kit, in mesangial cells from 8-week-old m/m mice treated with 0 (Vehicle) or 60 µM H_2_O_2_. Quantitative real-time PCR of *Tgfb1* and *Gapdh* mRNA in mesangial cells from 8-week-old m/m mice treated with 0 or 60 µM H_2_O_2_. Treatment with H_2_O_2_ significantly decrease DNA methylation and increase *Tgfb1* mRNA expression. Data represent ± SEM. n = 6. (**E**) The effect of siRNA targeting *Usf1* on upregulation of *Tgfb1* mRNA by reactive oxygen species. Quantitative real-time PCR of *Tgfb1* (normalized to *Gapdh*) mRNA in mesangial cells, from 8-week-old m/m mice, treated with 0 or 60 µM H_2_O_2_, and with control or *Usf1*-targeting siRNA. Transfection of *Usf1* siRNA abolish the upregulation of *Tgfb1* mRNA induced by H_2_O_2_, whereas control siRNA did not affect H_2_O_2-_induced increase in *Tgfb1* mRNA_._ Dunnett -corrected t-tests. Data represent the mean ± SEM. n = 6. (**F**) Quantitative real-time PCR of DNMT1 mRNA (normalized to *Gapdh*) in mesangial cells, from 8-week-old m/m mice, treated with 0 or 60 µM H_2_O_2_. H_2_O_2_.induced significant decrease in DNMT1 mRNA. Data represent the mean ± SEM. n = 6. (**G**) Quantitative real-time PCR of DNMT1 mRNA (normalized to *Gapdh*) and western blot analysis of DNMT1 (normalized to β-actin) from 15-week old *db/db* mice treated or not treated with Tempol for 8 weeks. Tempol tended to increase DNMT1mRNA and protein. Data represent the mean ± SEM. real-time PCR; n = 6, western blot analysis; n = 3. Original gel image of western blot analysis are presented in Supplemental Fig. 9.

There is accumulating evidence indicating that overproduction of ROS in the kidney can induce diabetic glomerular injury and mesangial fibrosis through TGFB1 upregulation in DN^26–28^. To investigate the effect of ROS on methylation of the *Tgfb1* gene, we performed treatment with anti-oxidants, Tempol in the drinking water of db/db mice. At first, to determine whether ROS was overproduced in *db/db* mice relative to controls, we measured urinary excretion of 8-OHdG and malondialdehyde (MAD) content in the kidney. Both the amount of 8-OHdG in the urine and renal MAD content were significantly increased in *db/db* mice compared with *db/m* controls (Fig. 6A). Moreover, treatment with Tempol significantly decreased these parameters. Of note, Tempol reversed the DNA demethylation (Fig. 6B) associated with the increased and decreased binding of DNMT1 and USF1 to their respective binding sites (Fig. 6C), resulting in a significant reduction of *Tgfb1* mRNA levels (Fig. 6B).

Given ROS-induced global hypo-methylation^38^, we investigated whether the inhibition of ROS with Tempol can modulate the binding of DNMT1 to the other genes than TGFB1, all of which genes are demethylated. Consistently, Tempol significantly increased its binding to the promoters of *TgfRII*, *Ctgf* and *Igf-1* and tend to increase to *Pdgf* gene promoter (Supplemental Fig. 7). These findings suggest that ROS induced global DNA hypomethylation in certain genes, via decreased binding activities of DNMT1.

### ROS induced DNA demethylation of the *Tgfb1* promoter in mesangial cells, *in vitro*

To investigate the direct effect of ROS on methylation of the *Tgfb1* gene, we performed an *in vitro* study using mesangial cells from misty (*m/m*) control mice. After addition of H_2_O_2_ to primary mesangial cells from control mice, the status of DNA methylation was analyzed. Treatment with H_2_O_2_ induced DNA demethylation of the *Tgfb1* promoter region in mesangial cells (Fig. 6D). RT-PCR analysis demonstrated that treatment with H_2_O_2_ led to an increase in *Tgfb1* mRNA in mesangial cells (Fig. 6D). To evaluate the involvement of USF1 binding to the promoter in the ROS-induced upregulation of *Tgfb1* mRNA expression, the effect of USF1 knock-down was examined. Transfection of mesangial cells with siRNA targeting *Usf1* led to >75% suppression of *USF1* mRNA and protein expression (Supplemental Fig. 8) and could ameliorate the upregulation of *Tgfb1* mRNA induced by H_2_O_2_ (Fig. 6E). Of note, treatment with H_2_O_2_ induced downregulation of DNMT1 in cultured mesangial cells (Fig. 6F); conversely, treatment with Tempol could reverse the decreased expressions of both DNMT1 mRNA and protein in mesangial cells of *db*/*db* mice (Fig. 6G and Supplemental Fig. 9).

### Tempol can improve mesangial fibrosis in the *db/db* mouse

**Figure 7:**
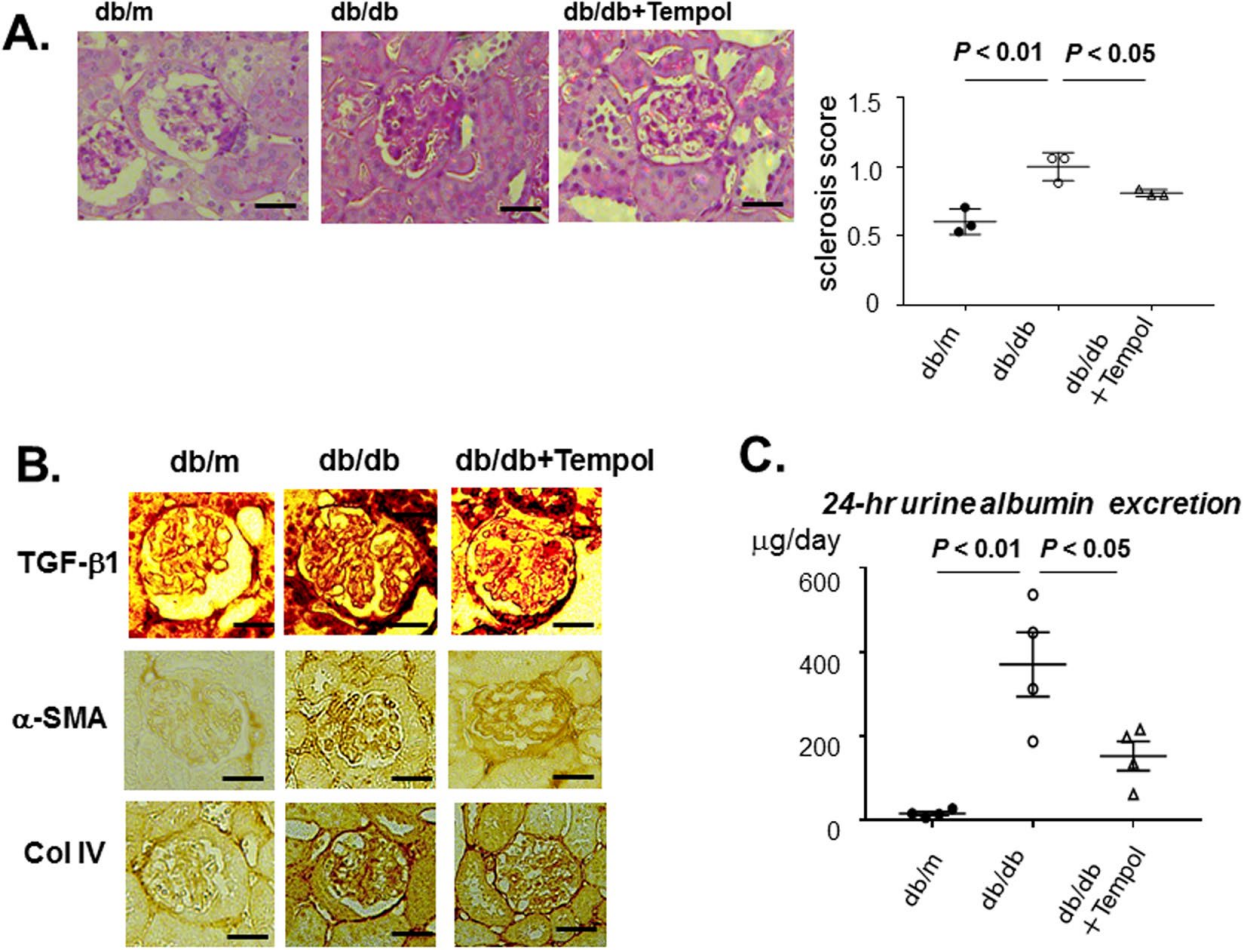
The effect of anti-oxidants on glomerulosclerosis in *db/db* mice. (**A**) PAS staining of glomeruli from 15-week-old *db/m*, *db/db*, and *db/db* mice treated with Tempol for 8 weeks. Scale bar, 20 µm. Mesangial sclerosis is significantly increased in *db/db* mice compared to *db/m* mice, but Tempol significantly decrease in *db/db* mice. Semi-quantitative glomerulosclerosis scores of 20 glomeruli each from three mice per group. Data represent the mean ± SEM. n = 3. Filled circle: *db/m* mice; open circle: *db/db* mice; open triangle open triangle: db/db+ Tempol. (**B**) Representative images of immunostaining of TGFB1, α−smooth muscle actin (α-SMA), and type IV collagen (Col IV) in glomeruli from 15-week-old *db/m*, *db/db*, and *db/db* mice treated with Tempol for 8 weeks. Treatment with Tempol attenuated increased immunostaining of TGFB1, α-SMA and Col IV in the glomeruli of *db/db* mice. Scale bar, 20 µm. (**C**) 24-hr urinary albumin excretion at 15-week old *db/m*, *db/db* and *db/db* treated with Tempol for 8 weeks. Urinary albumin excretion is significantly increased in *db/db* mice compared with *db/m* mice, but treatment with Tempol significantly reduce the increased urinary albumin in *db/db* mice. Data represent the mean ± SEM. n = 4.

Given the inhibitory effect of Tempol on *Tgfb1* mRNA expression, we examined the effects of treatment with this drug on mesangial fibrosis and matrix expansion. Periodic acid-Schiff (PAS) staining revealed that Tempol could ameliorate increased mesangial fibrosis and matrix expansion in *db/db* mice (Fig. 7A). Consistent with this result, increased immunostaining of TGFB1, α-smooth actin, and type IV collagen in the glomeruli of *db/db* mice was attenuated by treatment with Tempol (Fig. 7B). Finally, Tempol could ameliorate the increased urinary albumin excretion in *db/db* mice (Fig. 7C).

## Discussion

DN is a serious complication of diabetes and the primary cause of ESRD, and the number of patients with DN continues to increase, despite improved management of diabetes. Given the irreversibility of DN^3,4^, epigenetic mechanisms may be involved in the persistent phenotypic changes of blood vessels and organs^5,6^ that result in diabetes-related complications, including DN^7^. However, research on DNA methylation in DN is somewhat limited compared with that in other tissues affected by diabetes, such as blood cells^39^, skeletal muscle^24^, and liver^40^. Genomic DNA from peripheral whole blood of diabetic patients with nephropathy displayed differential methylation at several genes previously linked to DN relative to patients without DN^39^. Moreover, results from the DCCT/EDIC type 1 diabetes cohort study demonstrate that changes in DNA methylation status in circulating cells of diabetic patients persist for over 15 years, supporting an epigenetic explanation for metabolic memory^41^.

In the kidney, DNA methylation of *RASAL1*, which encodes an inhibitor of the RAS onco-protein, was found to be associated with epithelial-mesenchymal transition and fibrotic fibroblast activation in a model of renal fibrosis; however, DNA methylation in the whole kidney, including fibroblasts, was evaluated in that study^8^. Upregulation of DNA methylation and DNMT1 expression have been also observed in the kidneys of chronic kidney disease and diabetic kidneys^34,42,43^. Indeed, in the present study, the mRNA and protein expression of DNMT1 was significantly increased in whole kidney of db/db (Supplemental Fig. 4), as previously reported^34^. However, the DNA methylation status of whole kidney represents the sum of the methylation of all of the dozens of different cell types that compose the organ. DNA methylation is cell type-specific and differs among individual cells. In the present study, therefore, to evaluate the mesangial cell-specific DNA methylation status, we purified mesangial cells by culturing glomerular cells sieved from the kidneys of diabetic (db/db), and control non-diabetic (db/m), mice. As a result, we found downregulation of DNMT1 mRNA in mesangial cells, in contrast to increased expression in the whole kidney. Given that the kidneys have by far larger number of the other glomerular cells such as podocytes and tubular cells than those in mesangial cells, appearance of downregulated DNMT1 mRNA expressions in mesangial cells might be unmasked by purification. Supporting this, decreased mRNA expressions of DNMT1 observed in mesangial cells was absent in the glomeruli sieved from diabetic kidneys (Supplemental Fig. 5) . Consistently, either folic acid (Fig. 5E) or Tempol (Fig. 6G), could reverse the decreased DNMT1 mRNA expression in mesangial cells of diabetic mice, followed by increased DNA methylation of *Tgfb1* gene, and H_2_O_2_, *in vitro*, induced downregulation of DNMT1 mRNA in cultured mesangial cells (Fig. 6F), followed by decreased DNA methylation of its gene. Thus, the expressions of DNMTs might correlate with their binding activities on *Tgfb1* promoter, although it is still unclear how the downregulated expression of DNMT1 decreases its binding activity on *Tgfb1* promoter.

Recently, an examination of DNA methylation profiles in the micro-dissected tubular compartment, primarily consisting of proximal tubules including interstitial and vascular cells, from patients with chronic kidney disease and DN, and controls, revealed significant differences in their genome-wide DNA methylation, and key differentially methylated genes were related to fibrosis^44^. Regarding cell type-specific DNA methylation, our previous study showed that diabetes induces aberrant DNA methylation and a concomitant increase in angiotensinogen mRNA expression in proximal tubules purified by sorting from diabetic kidneys^10^.

In addition to its potential role in tubular injury during the progression of chronic kidney disease^45,46^, mesangial cell fibrosis is recognized as a common phenotypic change in progressive DN; however, DNA methylation studies on glomerular cells from diabetic kidneys are limited, due to the numbers of different cells, including podocytes, epithelial cells, and mesangial cells, in the glomerulus. Moreover, proximal tubular cells are known to invaginate into the mouse glomerulus, and TGFB1 is highly expressed in the parietal layer of Bowman’s capsule and tubule-interstitial cells, as well as in mesangial cells^47,48^. In the present study, however, neither other glomerular cells, nor proximal tubular cells, contaminated the primary cultures of cells generated from sieved glomeruli, despite the presence of glomerular cells in samples after sieving from the kidney, without subsequent culture.

Several investigators demonstrated that ROS induced either global hypomethylation or regional hypermethylation in certain genes. According to global hypomethylation induced by ROS^49–51^, we found hypomethylation of not only *Tgfb1* gene but also the other fibrosis-related genes such as *TgfRII*, *Ctgf* and *Igf-1* and *Pdgf* genes in primary culture mesangial cells of *db/d*b diabetic kidney. In accordance with ROS-induced regional hypermthylation^52,53^, however, DNA hypermethylation occurred in several ROS-producing genes including *Nox1* and *Txnip*, possibly by negative feedback of upregulated gene expressions which are reported previously in diabetic kidneys^54,55^. According to ROS-induced global hyomethylation, 8-OhdG decrease DNA methylation through the inhibition of DNMT-mediated cytosine methylation, in addition to a direct modification of DNA by 5-hydroxymethylcytosine (5hmC) which is the result of the hydroxylation of methylcytosine through attack by ROS. As a result, DNA demethylation of *Tgfb1*, *TgfRII*, *Ctgf*, *Pdgf*, *Igf-1*, *p65*, *Usf-1*, *At1a* and *At1b* may contribute to activation of these fibrosis-related genes, and result in mesangial fibrosis and matrix expansion. Among these fibrosis related genes, TGFB1 is known to play a critical role in mesangial fibrosis under diabetic conditions, we focused on aberrant DNA methylation of *Tgfb1* promoter genes.

Thus, aberrant DNA methylation and concurrent upregulation of *Tgfb1* mRNA reported here were observed in mesangial cells specifically, demonstrating the important role of mesangial cell-specific DNA demethylation in upregulation of *Tgfb1* mRNA expression and subsequent mesangial fibrosis and matrix expansion in DN. According to the causative relationship between DNA methylation and mRNA expression, moreover, the result of treatment of folic acid supported the crucial role of DNA demethylation in upregulation of *Tgfb1* mRNA expression, although folic acid induced global DNA methylation.

The epigenome, which includes covalent modifications of DNA and its transcriptional machinery, is the key determinant of outcome following transcription factor binding. Increased promoter methylation can interfere with transcription factor binding, causing loss of, or interference in, expression of specific genes. Conversely, decreased methylation increases gene expression. Moreover, DNA methylation status at the binding sites of specific transcription factors, rather than other promoter regions, is the main determinant of the binding of these proteins to promoters, leading to the transcription of specific genes. Notably, the present finding of increased USF1 binding to its recognition site in *Tgfb1* promoter region (−641 to −636 bp) and unchanged SREBP1 binding to its site (−220 to −32 bp) in *db/db* mice is consistent with the results of bisulfite sequence analysis revealing that the CpG dinucleotide at position −639 bp, within the USF1-binding site, was demethylated in *db/db* mice compared with *db/m* mice, while the CpG at −207, which neighbors the SREBP1-binding site was demethylated at comparable levels in both *db/m* and *db/db* mice. Thus, the DNA methylation status of the CpG at the USF1-binding site in the *Tgfb1* promoter determines USF1 binding and regulates transcription of the gene. This hypothesis is supported by our *in vitro* assays demonstrating that the transfection of siRNA targeting *Usf1* could reverse ROS-induced upregulation of *Tgfb1* mRNA in mesangial cells of *m/m* mice. Tempol treatment of *db/db* mice reversed the aberrant DNA methylation, and increased and decreased the binding of DNMT1 and USF1, respectively, at their recognition sites in *Tgfb1* promoter, decreasing *Tgfb1* mRNA expression. Taken together, these findings indicate that ROS-induced DNA demethylation of CpGs, and the consequent release of DNMT1 from the USF1-binding site in *Tgfb1* promoter, elevates binding of USF1 to its recognition site, leading to *Tgfb1* mRNA expression.

A number of different factors potentially contribute to the persistence and irreversibility of DN, although hyperglycemia is a primary cause of the condition. Certainly, transient exposure of endothelial cells to hyperglycemia *in vitro* causes epigenetic changes and altered gene expression, and both the epigenetic and gene expression changes persist during subsequent normo-glycemia^27,56^. These persistent epigenetic effects of transient exposure to hyperglycemia have also been demonstrated in animal models of diabetic kidney disease^3^. According to the plausible hypothesis that persistent changes in gene expression are largely mediated by epigenetic modifications^9^, diabetes-induced aberrant DNA methylation of target genes related to glucose metabolism and transport in the proximal tubules of the kidney leads to resistance to the effects of antidiabetic drugs^10^. In the present study, we consistently observed that changes in DNA methylation and mRNA expression persisted simultaneously in mesangial cells from *db/db* mice, reminiscent of the inevitable progression of DN. Indeed, early intervention to ensure good glucose control is required for reversal of persistent epigenetic changes; however, elevated glucose levels are not the sole factor contributing to maladaptive epigenetic modifications in diabetes. Epigenetic programming can also be influenced by periods of hyperglycemia, obesity, or other components of the diabetic milieu, which promote maladaptive activation of pathogenic pathways, even after such risk factors are belatedly treated.

Interestingly, in streptozotocin-induced diabetic rats with poor metabolic control for 13 months soon after induction, but not those with good control, overproduction of ROS was observed in the renal cortex and urine, although, when reinstitution of good control was delayed for 6 months after induction of diabetes, ROS overproduction remained elevated, with an insufficient reduction in albuminuria^3^. The finding that hyperglycemia-induced oxidative stress can be prevented if good control is initiated very early, but is not easily reversed if poor control is maintained for longer durations, led us to the hypothesis that transient exposure to hyperglycemia can cause persistent albuminuria during periods of normo-glycemia because of specific long-lasting epigenetic changes induced by ROS. Indeed, DNA methylation can be influenced by ROS, both directly through oxidative modification preventing DNA methylation and indirectly through its effects on methylation writing/erasing enzymes^25,26^.

Of note, epigenomic profiling of the DCCT/EDIC cohort study^40^ demonstrates differential methylation of TXNIP, which persisted for over 15 years in peripheral blood cells of type 1 diabetic patients^38^. Given the importance of ROS overproduction in diabetic glomerular injury and mesangial fibrosis through TGFB1 upregulation^16,28,29^, even delayed treatment with Tempol could decrease *Tgfb1* mRNA, inhibit mesangial cell fibrosis, and decrease albuminuria in diabetic rodents^30,31^. Consistently, treatment with the antidiabetic drug, pioglitazone, beginning at 6 weeks of age, failed to reverse aberrant DNA methylation of diabetic kidney-related genes in *db/db* mice^10^; however, even delayed treatment with Tempol, starting from 7 weeks of age, in the same diabetic mice with ROS overproduction could reverse both DNA demethylation of *Tgfb1* and concurrent upregulation of *Tgfb1* mRNA expression, with associated inhibition of mesangial fibrosis. These findings suggest that aberrant DNA demethylation, caused by ROS overproduction, has a critical role in the persistence of DN, which is somewhat resistant to the antidiabetic drug pioglitazone, but can be reversed by treatment with Tempol.

According to ROS generators, not only hyperglycemia but also angiotensin II is well known to increase ROS production. Given the presence of all the components necessary to generate intrarenal angiotensin II in both glomeruli and intra-tubular compartments^57^, local renin-angiotensin system is implicated in the progression of DN through ROS-induced activation of TGF-β signaling^58^. Moreover, treatment with the AT1 blockade, losartan, reversed histone modification profiles at genes associated with the pathology of DN in renal glomeruli of *db/db* mice^59^. The link between histone modification and DNA methylation^60^ leads to the plausible hypothesis that local RAS in mesangial cells may be involved in DNA demethylation and upregulation of *Tgfb1* mRNA in DN. However, further studies are needed to clarify the interaction between DNA methylation and histone methylation in persistent increase in *Tgfb1* mRNA expression induced by overproduction of RAS in DN.

In conclusion, the present study revealed DNA demethylation of *Tgfb1* in mesangial cells from diabetic kidneys and indicates the importance of cell type-specific analysis in the kidney. Aberrant DNA methylation in mesangial cells, caused by overproduction of ROS, underlies the persistent alterations of *Tgfb1* mRNA, likely leading to mesangial fibrosis in diabetic kidneys. In addition to the efficacy of the anti-oxidant therapy, investigation of epigenetic modifications, including DNA methylation, could pave the way for the development of novel therapeutic approaches to prevent and reverse the progression of DN.

## Methods

### Ethics statement

Animal care and treatment complied with the standards described in the Guidelines for the Care and Use of Laboratory Animals of the University of Tokyo. All methods were carried out in accordance with relevant guidelines and regulations of the University of Tokyo. All experimental protocols were approved by Animal Care and Use Ethics Committee of the University of Tokyo.

### Animals

Male C57BLKS/J misty control *m/m*, *db/m*, and *db/db* mice were purchased at 7–8 weeks old, from Japan SLC (Shizuoka, Japan). Animal care and treatment with NIH Guide for Care and Use of Laboratory Animals or the equivalent. To evaluate the effects of DNA demethylation and reactive oxygen species, folic acid (Wako Pure Chemical Industries, ltd, Japan) and Tempol (Merck Millipore ltd, Canada) was administered to 7-week-old *db/db* mice for 8 weeks. Folic acid and Tempol was mixed with drinking water and administered at a dose of 8 mg/kg/day and 2 mmol/l.

### Cell Culture

Primary mesangial cells were established from each model mouse using a sieving technique, which is described in detail in a previous report, with minor modification^32^. Cellular outgrowths from glomeruli were reached confluence approximately 10 to 14 days later, and cells were harvested and analyzed.

### DNA methylation analysis

DNA methylation analysis of fibrosis related genes promoter was performed using a MethylCollector Ultra kit (Active Motif, CA) according to the manufacturer’s protocol. Real-time PCR was performed using the resulting supernatant as template for specific primers to amplify regions flanking the fibrosis related genes TSS, including the promoter.

### Analysis of mRNA Levels

For quantitative RT-PCR analysis, cDNA template was generated using a QuantiTect Reverse Transcription Kit (Qiagen). The expression levels of mouse *Tgfb1* and *Gapdh* were then analyzed using the power SYBR Green PCR Master Mix (Applied Biosystems).

### Analysis of protein Levels

The protein levels of DNMT1, TGFb1, USF1 and SREBP1 were analyzed by immunoblotting using anti-DNMT1 antibody (Abcam, no. ab188453), anti-TGFb1 antibody (Abcam, no. ab92486), anti-USF1 antibody (Santa Cruz Biotechnology, no. sc-229) and anti-SREBP1 antibody ((R&D Systems, no. NB100-60545SS)). Expression levels of beta-actin were used for normalization. The protein level of beta-actin was analyzed using anti-beta-actin antibody (Abcam, no. ab8227). Wide-view prestained protein size marker III was used as size marker of DNMT1 protein (Wako, no. 230-02461).

### Bisulfite sequence analysis

Bisulfite conversion of 150–500 ng of genomic DNA was performed using the EpiTect Bisulfite kit (Qiagen). For bisulfite sequencing of the *Tgfb1* promoter, PCR products were cloned into the TA-cloning vector using a TOPO TA Cloning Kit (Invitrogen), and sequenced.

### ChIP assays

ChIP assays were performed using a Simple Enzymatic ChIP assay kit (Cell Signaling Technology), according to the manufacturer’s protocol. ChIP assays were performed using the following antibodies: mouse monoclonal anti-DNMT1 (Abcam, no. 92453); mouse monoclonal anti-DNMT3A (Abcam, no. 113430); goat polyclonal anti-DNMT3B (Abcam, no. 13604); rabbit polyclonal anti-USF1 (Santa Cruz Biotechnology, no. sc-229); and rabbit polyclonal anti-SREBF1 (R&D Systems, no. NB100-60545SS).

### Stimulation with H_2_O_2_ and Transfection of siRNA Targeting USF1

Primary mesangial cells were plated on 6 cm collagen I coated culture dishes. Three days later, 60 μM H_2_O_2_ was added, and 7 days later the culture medium was changed for fresh medium containing the same concentration of H_2_O_2_ plus 100 pmol of one of three siRNAs targeting mouse *Usf1* or 300 pmol of negative control siRNA (Qiagen), using 30 μl of Lipofectamine RNAiMAX reagent (Invitrogen). Seventy-two hours after transfection, mRNA was extracted and analyzed. The efficiency of siRNA knock-down of USF1 was confirmed by immunoblotting and quantitative real-time PCR.

### Detection of ROS

Twenty-four hour urine samples were collected from each mouse in metabolic cages. Levels of 8-OHdG in urine were investigated using a New 8-OHdG Check ELISA kit (Nikken Seil, Co., Ltd. Shizuoka, Japan). The reaction product of lipid peroxide in mouse kidney, MDA, was analyzed by immunoblotting using anti-MDA antibody (Nikken Seil, Co., Ltd. Shizuoka, Japan).

### Immunostaining analysis

Mesangial primary cells were fixed with 4% formaldehyde for 10 min, and immunohistochemistry was performed with goat polyclonal anti-alpha-8 integrin antibody (R&D Systems, no. AF4076), rabbit polyclonal anti-S100A4 antibody (Cell Signaling Technology, no. 13018), rabbit polyclonal anti-podocin antibody and mouse monoclonal anti-Na-K ATPase antibody (Santa Cruz Biotechnology, no. sc-21712). To confirm that primary culture cells do not contain proximal tubular cells, immunofluorescence staining was performed with FITC conjugated lotus tetragonolobus lectin. To confirm the specificity of the anti-alpha-8 integrin antibody and anti-S100A4 antibody, immunofluorescent staining of C57BL/6 J mice kidney fixed with 4% paraformaldehyde was performed. To evaluate the fibrosis of glomeruli in *db/db* mice, immunohistochemistry was performed with rabbit polyclonal anti-TGF-beta (Abcam, no. 92486), mouse monoclonal anti-smooth muscle actin (DAKO, no. M0851), and rabbit polyclonal anti-type IV collagen (R&D Systems, no. NB120-6586) antibodies

### Scoring of glomerulosclerosis

To evaluate the effect of Tempol on glomerulosclerosis, scoring of glomerulosclerosis was performed. PAS staining of 15-week-old kidneys from *db/m* and *db/db* mice with or without Tempol treatment was performed on paraffin-embedded tissue fixed with 4% paraformaldehyde for 2 h. Twenty glomeruli were graded for each kidney specimen according to the extent of glomerulosclerosis, using a previously described method^61^.

### Measuring of urinary albumin excretion

24-hr urinary albumin excretion of *db/m* and *db/db* mice with or without Tempol treatment was investigated with Mouse Albumin ELISA KIT (AKRAL-121, Shibayagi Gunma, Japan) with according to the manufacturer’s protocol.

### Statistics

Data are presented as means ± SE for at least three separate experiments. Mann-Whitney U test were used for statistical comparisons. ANOVA and Dunnett -corrected t-tests were used for multiple-group comparisons. Single star mark indicated p < 0.05 and double star marks indicated 0.05 < p < 0.10 between two groups. Values of p < 0.05 were considered statistically significant.

## Electronic supplementary material

Supplemental Figure 1–9 and Table
