# Supplementary material for: Aberrant DNA methylation of Tgfb1 in diabetic kidney mesangial cells
Source: Sci Rep. 2018 Nov 5;8:16338. doi: 10.1038/s41598-018-34612-3 (PMC6218490; doi:10.1038/s41598-018-34612-3)
Supplement: Supplementary file 1 — Supplemental Figure 1–9 and Table [file 41598_2018_34612_MOESM1_ESM.pdf]

Aberrant DNA methylation of *Tgfb1* in diabetic kidney mesangial cells

Shigeyoshi Oba[1], Nobuhiro Ayuzawa[1], Mitsuhiro Nishimoto[1], Wakako Kawarazaki[1], Kohei Ueda[1], Daigoro Hirohama[1], Fumiko Kawakami-Mori[2], Tatsuo Shimosawa[3], Takeshi Marumo[1], Toshiro Fujita[1]

1. Division of Clinical Epigenetics, Research Center for Advanced Science and Technology, The University of Tokyo, Tokyo, Japan
2. Division of Endocrinology, Mitsui Memorial Hospital, Tokyo, Japan
3. Department of Clinical Laboratory, International University of Health and Welfare, School of Medicine, Mita Hospital IUHW, Tokyo, Japan

Corresponding author

Toshiro Fujita, MD, PhD

Division of Clinical Epigenetics, Research Center for Advanced Science and Technology (RCAST), The University of Tokyo, Tokyo, Japan

E-mail: Toshiro.FUJITA@rcast.u-tokyo.ac.jp

Phone: +81-3-5452-5070

Supplemental Figure legends

Supplemental Figure 1

Quantitation of DNA methylation, using a Methylcollector Ultra kit, in mesangial cells of 15week age *db/m* control and *db/db* mice. The promoter region was demethylated at positions TSS -366, +121 and +181 in *db/db* mice compared to *db/m* mice. Data represent the mean±SEM. n=6. Filled circles: *db/m* mice; open circles: *db/db* mice.

Supplemental Fig.1

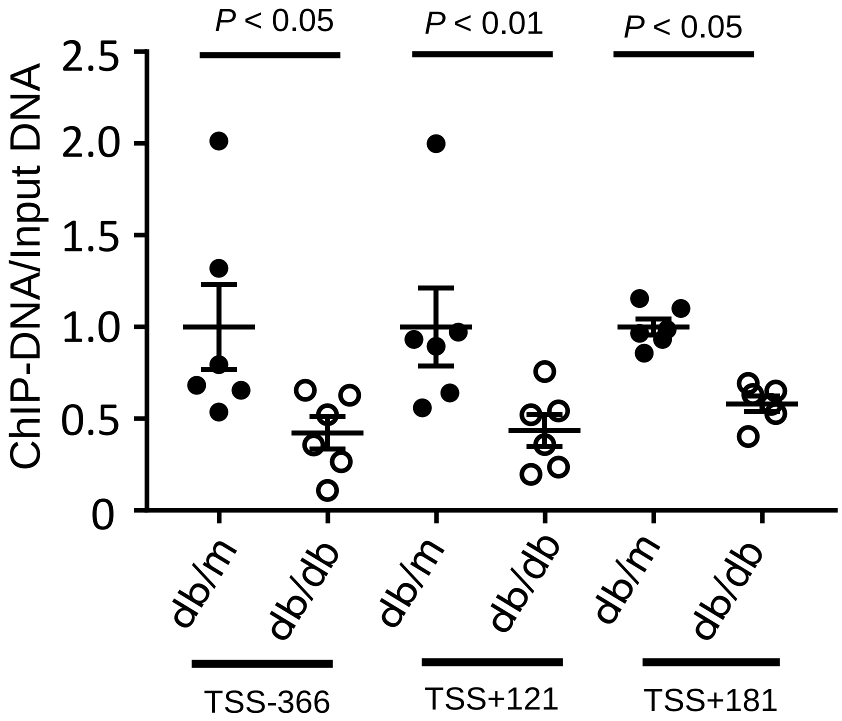

Supplemental Figure 2

DNA methylation of just sieving sample show comparable between db/db and db/m mice. Data represent the mean±SEM. n=6. Filled circles: db/m mice; open circles: db/db mice.

Supplemental Fig.2

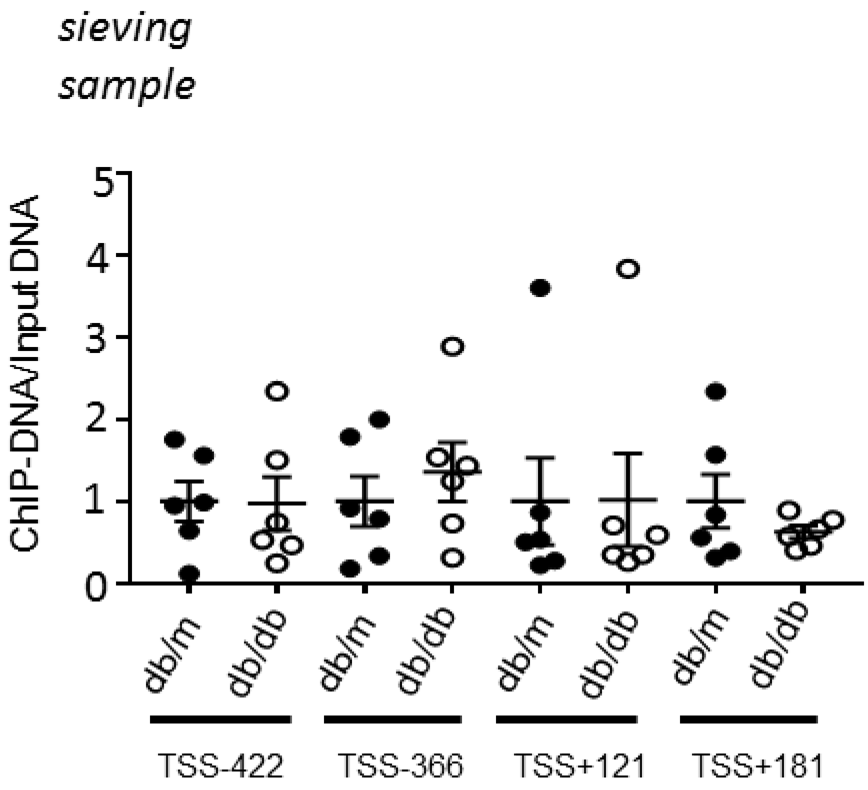

Supplemental Figure.3

Original image for manuscript Fig.4F

Supplemental Fig.3

Fig.4F

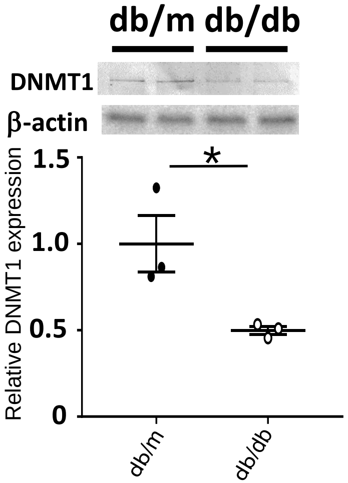

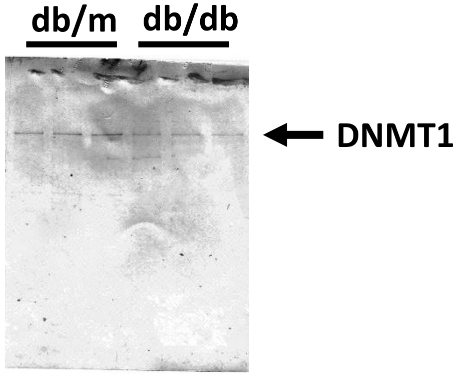

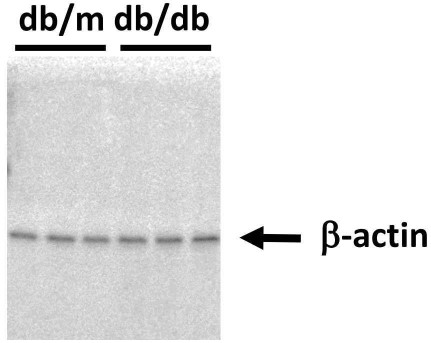

Supplemental Figure.4

Wester blot analysis and real-time PCR of DNMT1 of whole kidneys in db/m control and db/db mice. Data represent the mean±SEM. protein; n=3, mRNA; n=6.

Supplemental Fig.4

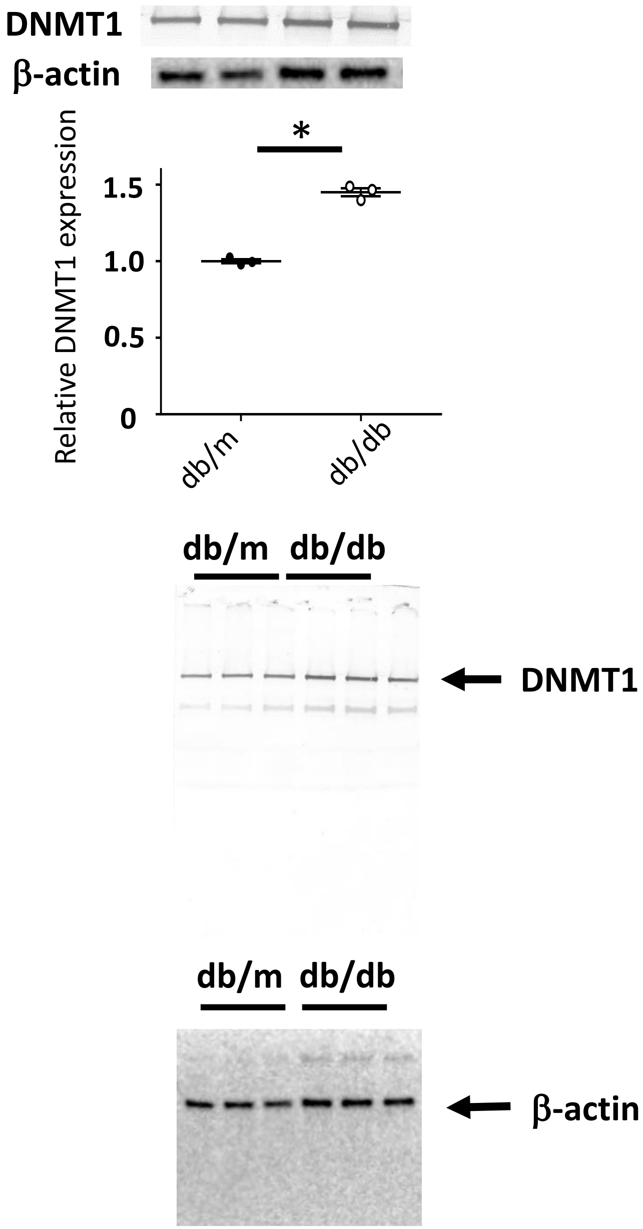

Supplemental Figure.5

Real-time PCR of DNMT1 of just sieving sample show comparable between dn/db and db/m mice. Data represent the mean±SEM. n=6. Filled circles: db/m mice; open circles: db/db mice.

Supplemental Fig.5

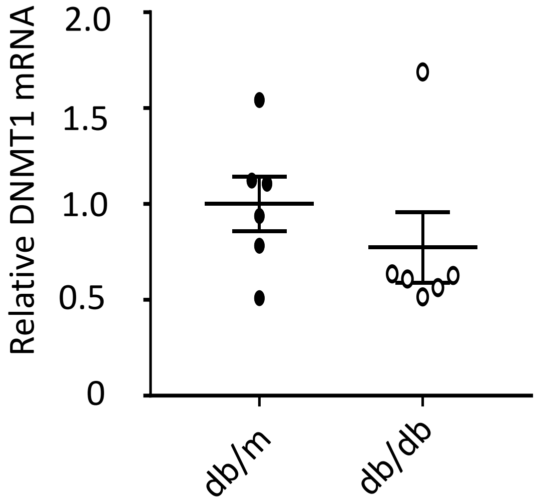

Supplemental Figure.6

Real-time PCR of Usf1 and Srebp1 mRNA in mesangial cells and immunoblotting of USF1 and SREBP1 expression in kidneys of db/db mice. Data represent the mean±SEM. mRNA; n=6, protein; n-3

Supplemental Fig.6

A.

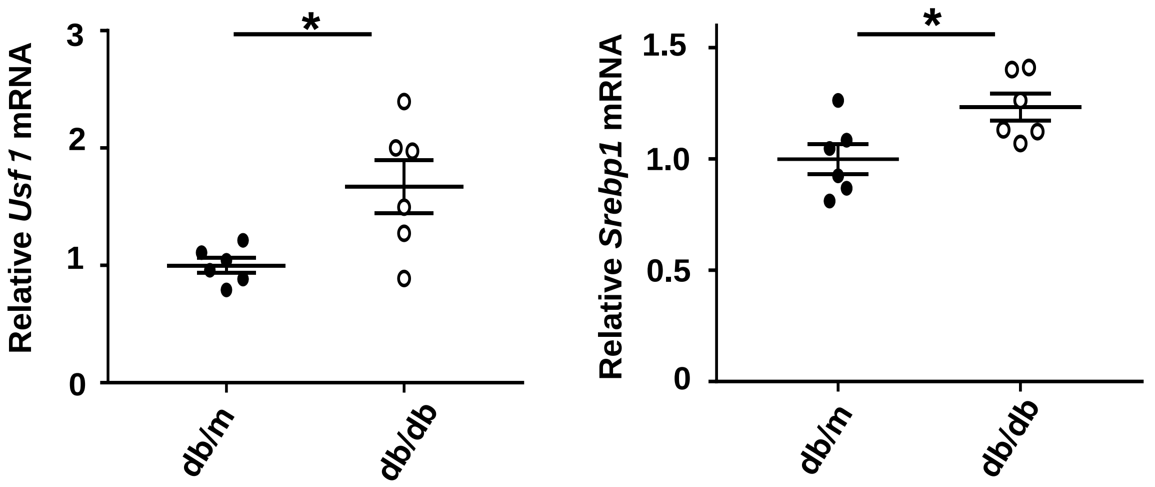

B.

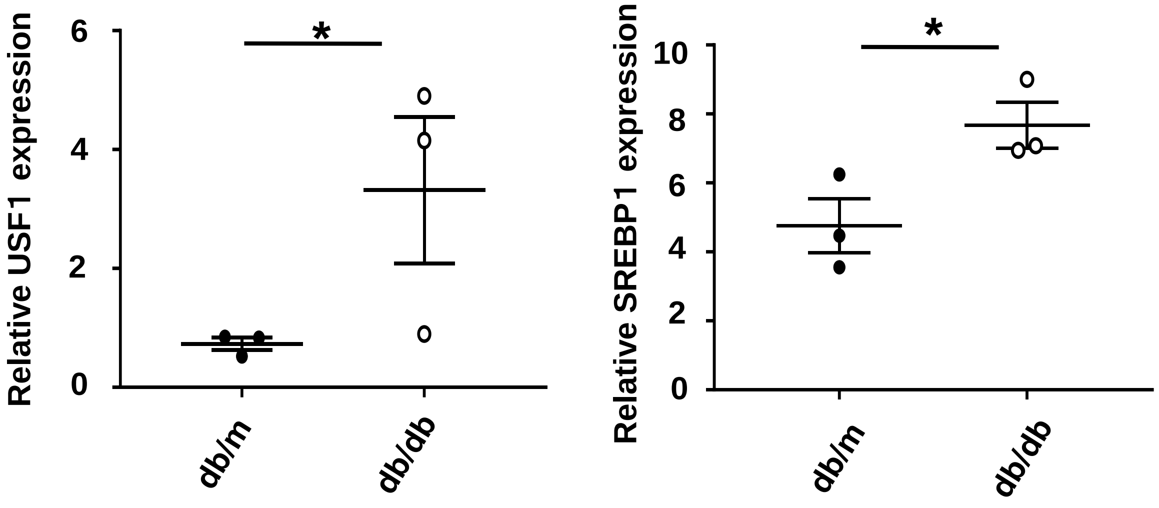

Supplemental Figure.7

The results of ChIP assays to determine binding of DNMT1 to the T gfbRII , C tgf , Pdgf and Igf - 1 promoter in mesangial cells from 15-week-old db/db mice treated or not treated with Tempol for 8 weeks. Tempol significantly increases the binding to TgfRII , Ctgf and Igf - 1 promoter and tends to increase to Pdgf promoter. Data represent the mean±SEM. n=6

Supplemental Fig.7

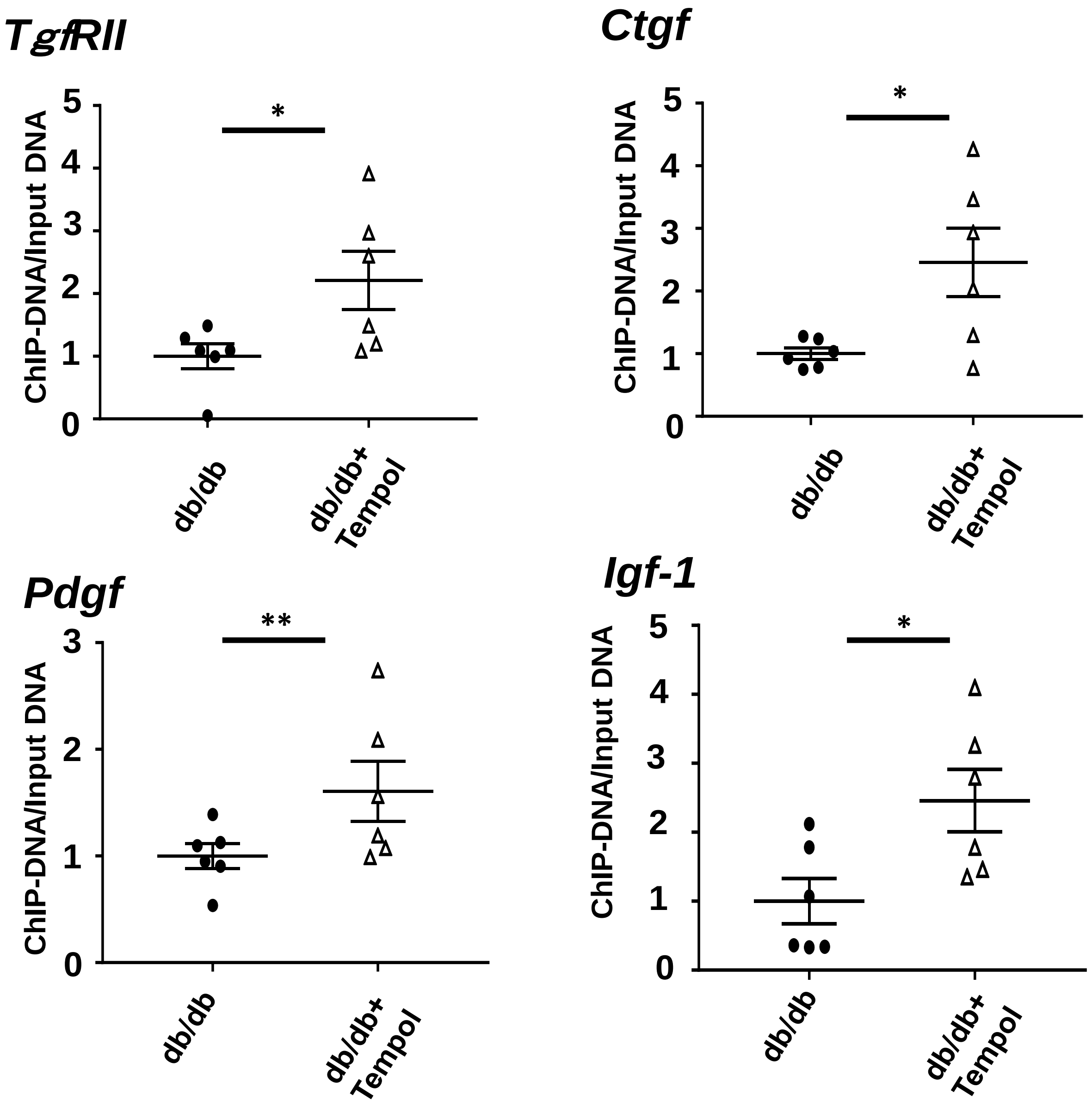

Supplemental Figure 8

The efficiency of knock-down of Tgfb1 mRNA and protein evaluated by real time PCR and immunoblotting of m/m mouse mesangial cells treated with control or Usf1 siRNA for 3 days. Data represent the mean±SEM. mRNA; n=6, protein; n=3.

Supplemental Fig. 8

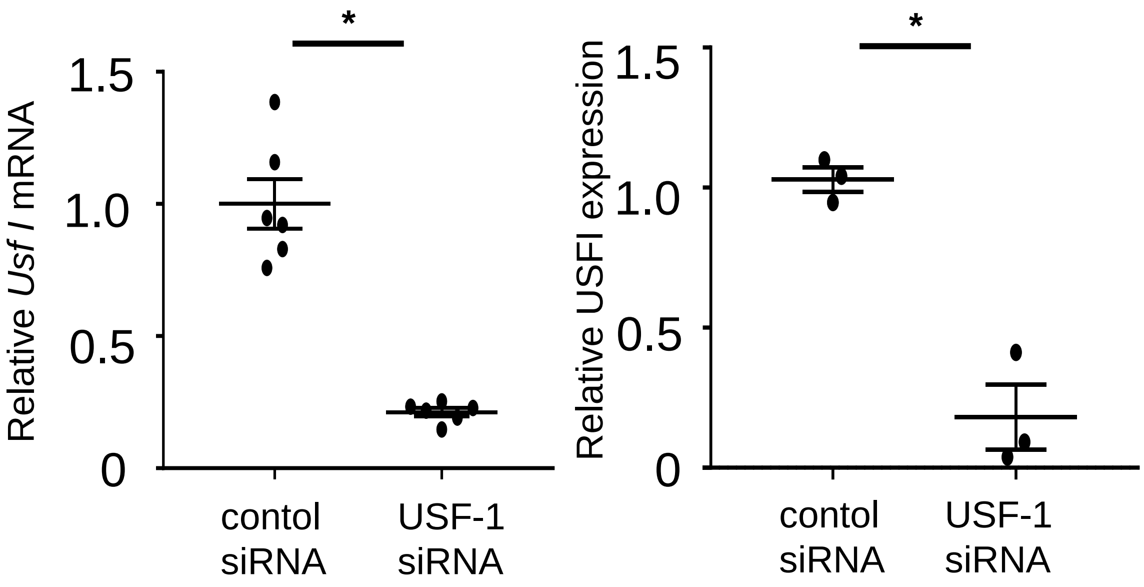

Supplemental Fig.9

Original image for manuscript Fig.6G

Supplemental Fig.9

Fig.6G

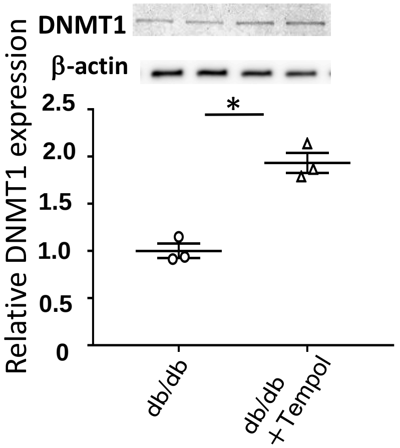

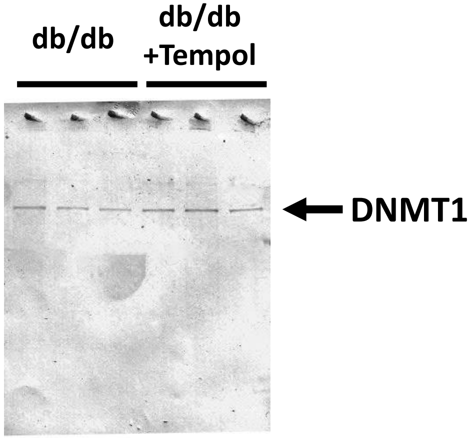

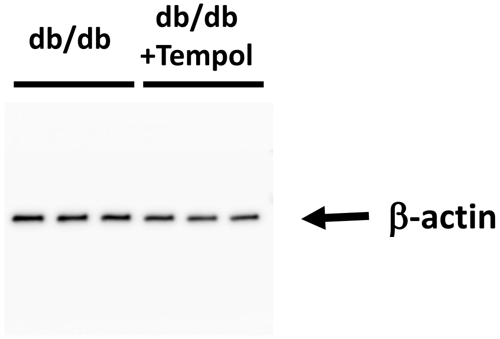

Supplemental Table

Template for specific primers to amplify regions flanking the *fibrosis related genes* TSS, primers for real-time PCR, and sequences of mouse USF1 siRNA

Mouse *Tgfb1*
TSS+121 primers
Forward Primer CTGAGCCGCACTCGGAGT
Reverse Primer CGGCTCCTCGGCTGCTC

TSS+181
Forward Primer TCCGAGGCCCCAGAGTCT
Reverse Primer CCACTCCTCCTCCCCCTC

TSS-366
Forward Primer GACACTCTCATCCGCAAAGT
Reverse Primer GGACCTAGGCGGTGGAGA

TSS-422
Forward Primer GTAAGGTGCCTCCTTGTATC
Reverse Primer AGGAGAGTGAAATAAGGAGAC

TSS+500 Forward Primer CACCCCAAAGCTGGGGCG
Reverse Primer GGGACTCAAGAGGCAGGCT

TSS-639
Forward Primer GAGCACATAATGGCACAGCA
Reverse Primer TGATCAGGACTGACAGTCTC

Mouse *TgfbRII*
TSS-170 primers
Forward Primer AAGTTCAAACTCAGAAGTGACT
Reverse Primer CAAGGAAGTGGTTTACACCA

Mouse *Ctgf*
TSS-155

Forward Primer CATCAGGAGGGGTGAGAAG
Reverse Primer CGCTGTGTTTCCAGCACAAC

Mouse *Pdgf*
TSS-780
Forward Primer AGCTACATCCATCCAAGCTC
Reverse Primer TTGCAGCTGGCACTGAAGG

Mouse *Igf-1*
TSS-289
Forward Primer AGAGGTCAGCTGCCTAGAG
Reverse Primer CCCTGGCTCAGGCACTGG

Mouse *Igf-2*
TSS-60
Forward Primer TTGTCTGATTTGCGGTCCTG
Reverse Primer CAAATTTAGTCATTCGCCACAG

Mouse *Igf binding protein 1*
TSS-285
Forward Primer GTGGGCATTGTCACGGAGA
Reverse Primer TCACAGAAAACCTAAGCGACT

Mouse *Igf R*
TSS-367
Forward Primer AACCGCTCATTCATTTCCACT
Reverse Primer GACGCAGTTGGCGAGACTG

Mouse *Smad 2*
TSS-207
Forward Primer AGTAGTGACACCAGGAAGAAA
Reverse Primer CGGATTCAGGAAGTAACATCT

Mouse *Smad 3*
TSS-400
Forward Primer CACAGACTCTCAGATACCGT

Reverse Primer GCCCTTGACGGACAGTGC

Mouse *Glut 1*
TSS-333
Forward Primer TTCAGCTTCGGAGCCAGAC
Reverse Primer ATGGTGTTTACAACCGCGTG

Mouse *Glut 2*
TSS-580
Forward Primer CCAGTTAGAGCATTATAGATGT
Reverse Primer GTCAGTGTATCTCAAGATGAG

Mouse *Col IV*
TSS-471
Forward Primer TGGGACAGAACTTTGGTCAC
Reverse Primer GTCATCTTAGCACCCTGACT

Mouse *Fibronectin*
TSS-255
Forward Primer TTTGCGGTCGTCAAACTTTTG
Reverse Primer CCTTCGAGTCTACATCACTTT

Mouse *α-sma*
TSS-261
Forward Primer CAAATGCCCAGACACTGGG
Reverse Primer ACAGGGGCTGGCATCTTTC

Mouse *Pai-1*
TSS-518
Forward Primer GGTTCTCGTTCCTCTTGGAC
Reverse Primer CTTCACAGCAGGCCTGGC

Mouse *Mmp-2*
TSS-179
Forward Primer CAGCGTCCTGATTCCAGTAT
Reverse Primer TGCGAGTCCTGAAGACTCTT

Mouse *Mmp-9*
TSS-280
Forward Primer GTCTCGGGCCTCAGGTCT
Reverse Primer ACCTAGCCCTAGGTCTGAC

Mouse *Vcam-1*
TSS-884
Forward Primer GACTATAAAACCTGCAGCATC
Reverse Primer AACTACATTCAGTATTTTGAACG

Mouse *Icam-1*
TSS-380
Forward Primer CAGGACTTGATTTCGGATCC
Reverse Primer CTCAAAGGGATAAACTGAGGC

Mouse *Nox-1*
TSS-675
Forward Primer CCTGCAGAATGCTTCCAGAC
Reverse Primer CCACTGAAGCTCTCCTTAGC

Mouse *Nox-4*
TSS-240
Forward Primer CCATTCTAGGAAGTAGGGTG
Reverse Primer AATGGGAATCGGAAGCTTGG

Mouse *Txnip*
TSS-476
Forward Primer GGGCTCTTTCTCACTAACACG
Reverse Primer ACAATCCCATCGCACCGAG

Mouse *Rage*
TSS-64
Forward Primer CTCAGGTCGGGTGAGATTG
Reverse Primer CTCGTTCTGTCAGAGAATGC

Mouse *p65*

TSS-281
Forward Primer AGGTGTCTGCCTAGTCCTC
Reverse Primer GCAGGAGTGCTTCGCGGA

Mouse *Il-6*
TSS-340
Forward Primer TCTGTCATGCGCGCGTGC
Reverse Primer CAATGTTTTATAAGGAGAAAGAG

Mouse *Mcp-1*
TSS-150
Forward Primer CGCAGCTTCATTTGCTCCC
Reverse Primer TGGAAGAGAGAAACTGTGGG

Mouse *Usf-1*
TSS-350
Forward Primer GGTCTTTGGAGTATTGCTGAC
Reverse Primer GGACATTTCTGAGTGTACGTG

Mouse *Srebp-1*
TSS-150
Forward Primer CTGGGCCTTGGCTTCTTCT
Reverse Primer CCCTTTAATCTAACGATGTCTG

Mouse *At1a*
TSS-180
Forward Primer GTTCATGTGGCCTTACGCC
Reverse Primer GATGTGCCCTTCCAGATCC

Mouse *At1b*
TSS-523
Forward Primer CATTACAACTATTCTTGTTAACG
Reverse Primer GGATACAAGTCAAGAGACCG

Mouse *Tgfb1*
Forward Primer CAAACTAAGGCTCGCCAGTC

Reverse Primer GTTGTACAAAGCGAGCACCG

Mouse *Gapdh*
Forward Primer GACCCCTTCATTGACCTCAAC
Reverse Primer CTTCTCCATCGTGGTGAAGA

Mouse *Usf-1*
Forward Primer CTGCCACTCAGTCTATGACC
Reverse Primer ACGGCATCGTCACTGGTGAA

Mouse *Srebp1*
Forward Primer TGGACGAGCTGGCCTTCG
Reverse Primer TCAAAACCGCTGTGTCCAGT

Mouse DNMT1
Forward Primer CCAAGCTCCGGACCCTGGATGTGT
Reverse Primer CGAGGCCGGTAGTAGTCACAGTAG

Mouse DNMT3a
Forward Primer GCACCTATGGGCTGCTGCGAAGACG
Reverse Primer CTGCCTCCAATCACCAGGTCGAATG

Mouse DNMT3b
Forward Primer CAAGGAGGGCGACAACCGTCCATT
Reverse Primer TGTTGGACACGTCCGTGTAGTGAG

mUSF-1 siRNA-1
Sense: GGACCCAACUAGUGUAGCUtt
Antisense: AGCUACACUAGUUGGGUCCtt

mUSF-1 siRNA-2
Sense: CCAGCACAGGUCAAUUCUUtt
Antisense: AAGAAUUGACCUGUGUGGtt

mUSF-1 siRNA-3
Sense: GCCGGGACAAGAUCAACAAtt

Antisense: UUGUUGAUCUUGUCCCGGCtt
